# Clinical Impact and Cost-Effectiveness of an Education Program for PD Patients: A Randomized Controlled Trial

**DOI:** 10.1371/journal.pone.0162646

**Published:** 2016-09-29

**Authors:** Cindy Canivet, Nadège Costa, Fabienne Ory-Magne, Céline Arcari, Christine Mohara, Laure Pourcel, Hélène Derumeaux, Emilie Bérard, Robert Bourrel, Laurent Molinier, Christine Brefel-Courbon

**Affiliations:** 1 Department of Neurology, University Hospital, Toulouse, France; 2 Department of Medical Information, University Hospital, Toulouse, France; 3 INSERM UMR1027, Toulouse, France; 4 Unité de pharmacoépidémiologie, Faculté de Médecine, Toulouse, France; 5 Department of Clinical Pharmacology, University Hospital, Toulouse, France; 6 Inserm, Imagerie cérébrale et handicaps neurologiques, UMR 825, F-31059 Toulouse, France; 7 Département d’Epidémiologie, Economie de la Santé et Santé Publique, UMR 1027 INSERM, Université de Toulouse III, Centre Hospitalier Universitaire de Toulouse, Toulouse, France; 8 Direction Régionale du Service du Contrôle Médical, Assurance Maladie, Toulouse, France; University of Toronto, CANADA

## Abstract

**Background:**

Parkinson’s disease (PD) is characterized by its impact on quality of life, constituting a substantial economic burden on society. Education programs implicating patients more in the management of their illness and complementing medical treatment may be a beneficial adjunct in PD. This study assessed the impact of an education program on quality of life and its cost-effectiveness in PD patients.

**Methods:**

This single-center, prospective, randomized study assessed an education program consisting of individual and group sessions over a 12-month period. A total of 120 PD patients were assigned to either the Treated by Behavioral Intervention group (TTBI) or the no TTBI group. The primary outcome criterion was quality of life assessed using PDQ39. The Unified Parkinson’s Disease Rating Scale (UPDRS) and psychological status were collected. An economic evaluation was performed, including calculations of incremental cost-effectiveness ratios (ICERs).

**Results:**

After 12 months of follow-up, changes recorded in the PDQ39 between the groups were not significantly different but better changes were observed in each dimension in the TTBI group compared to the no TTBI group. UPDRS I, II and total score were significantly improved in TTBI group compared to the no TTBI group. Mean annual costs did not differ significantly between the two groups.

**Conclusion:**

This study suggested that the education program positively impacts the perceived health of PD patients without increasing medical costs.

## Introduction

The prevalence of Parkinson’s disease (PD) in Europe is approximately 160/100,000 among individuals aged 65 years and over, and the number of cases is expected to increase considerably in the coming years [1]. This complex disorder is characterized by motor signs, but also by an impressive diversity of non-motor symptoms, including fatigue, sleep disorder, pain, depression, anxiety and so on, often occurring simultaneously in the same patient. Several studies using quality of life (QOL) instruments have shown that PD affects health-related QOL and that patients have higher levels of distress than healthy elderly people [2–3]. Nearly half of PD patients use at least one alternative therapy [4]. Education programs allowing patients to acquire knowledge and skills relating to their disease and to become more implicated in its management, as a complement to medical treatment, may be a beneficial adjunct in PD.

Education programs have been implemented in several countries in Europe and in the United States and have provided evidence supporting PD patient education [5–11]. However, there have been only a few studies addressing the effectiveness of PD patient education based on a strong methodology [7–8; 10; 12–13]. These studies reported improvements in QOL, psychological well-being and compliance with drug treatment.

PD represents a substantial economic burden on society because patients are less able to work, and require care and costly treatment [14]. Several economic studies have calculated the annual direct costs of PD [15], but none has yet assessed the cost-effectiveness ratio associated with an education program for PD patients.

The goal of this randomized prospective controlled pilot study was to assess the impact of an education program on QOL, and motor and psychological functions and to assess the cost-effectiveness of a therapeutic education program, compared to traditional care in PD patients.

## Methods

### Patients

This pragmatic study was based on a sample of PD patients selected from the movement disorders outpatient clinic of the Neurological University Hospital of Toulouse (France). Consecutive patients with idiopathic PD according to the United Kingdom Parkinson’s Disease Society Brain Bank Clinical Diagnosis Criteria were invited to participate in the study. For inclusion, PD patients had to fulfill the following criteria: (1) treated with antiparkinsonian drugs and/or by deep brain stimulation of the subthalamic nucleus (if surgery had occurred at least six months previously), (2) had a Hoehn and Yahr scale score of ≥4, (3) had a Mini-Mental State Examination score > 25/30, (4) was able to complete a self-report questionnaire. For each patient, chronic conditions were assessed and classified into broad disease categories, such as cardiovascular diseases, tumors, respiratory diseases, metabolic diseases, according to the 10^th^ revision of the International Disease Classification (ICD-10). This research was approved by the French ethics committee: “Comité de protection des personnes Sud Ouest et Outre-Mer II” number 02-08-22. All patients gave their written informed consent for the protocol and for the medical cost collection. This trial is registered with ClinicalTrials.gov under number NCT01717144. The clinical trial registration was delayed because the sponsor of the study did not consider this work as a clinical trial (as recommended by the FDAAA 801) due to lack of drugs, biologics or devices but as a behavioral intervention. This manuscript reflects perfectly the initial methodology of this work.

### Intervention: education program

The education program consisted of quarterly 90-minute individual sessions with the PD education nurse and three four-hour group sessions (with a maximum of 10 patients). Each group session was run by at least two healthcare professionals (physician, nurse, physiotherapist, speech therapist, or psychologist). For each patient, the topic of the individual sessions was based on an educational assessment of the patient’s needs and environment (educational diagnosis) and on the learning priorities identified by the patient and healthcare providers, including self-monitoring techniques, such as keeping a diary, improving compliance with drug treatment, identification of non-motor symptoms, maintaining mobility, and improving well-being [16]. The group sessions consisted of thematic workshops, focusing on topics such as physical activity, stress management, social support, language and communication. The education team provided the patients with information and worked with them to develop strategies to implement at home, using tools and communication techniques. This education program ran for 12 months. At the end of the program, an assessment highlighted the skills gained by the patient with the aim of updating the education goals. During the end-of-study visit, patients completed a satisfaction questionnaire on a scale of 0 to 10 on the education program and were asked to indicate whether they would like to continue with the program.

### Study design and assessment criteria

A single-center, prospective, randomized study was carried out. PD patients were assigned by a computer-generated randomization sequence to either the intervention group (Treated by Behavioral Intervention, TTBI): immediate education program in addition to usual neurological care, or the no TTBI group: delayed education program where these patients were offered only the standard neurological care for 12 months and were then asked whether they wished to participate in the education program. Randomization was balanced by blocks of six patients. The randomization codes were kept in the pharmaco-epidemiological unit.

Clinical outcomes and characteristics of patients were assessed just before randomization, and again six months and 12 months later.

The primary evaluation criterion was QOL assessed by the Parkinson’s Disease QOL PDQ-39 [17]. The PDQ-39 contains 39 items covering eight dimensions: mobility, activities in daily life, emotional well-being, stigma, social support, cognitions, communication and bodily discomfort.

The generic QOL questionnaire SF36 was also used: the Medical Outcomes Study 36-item Short Form SF-36, which has eight scales: physical functioning, role limitations due to physical health, bodily pain, general health perceptions, vitality, social functioning, role limitations due to emotional problems, and mental health.

Motor status was assessed with the Unified Parkinson’s Disease Rating Scale (UPDRS, Part III-motor examination). The UPDRS Part I-mental, behavior and psychological state; Part II-activities of daily living (ADL) and Part IV-side effects of PD treatment were also collected. Patients were assessed in ON status (under their usual dopaminergic treatment ± ON STIM for patients with deep brain stimulation).

Patients psychological status was evaluated using the Hospital Anxiety and Depression Scale (HADS).

An economic evaluation (from the healthcare payer’s perspective (i.e. the national social health insurance) was performed over one year, taking into account direct medical and non-medical costs. Direct medical costs included medication, hospitalizations, outpatient care (i.e. medical visits, complementary examinations) and direct non-medical costs were limited to transportation costs. Contrary to the initial protocol, instead of collecting health consumption through a patient notebook, the data used were collected retrospectively from the French social health insurance databases and included only PD patients covered by it. A bottom-up approach was used to collect resources utilization.

The cost of hospitalization was assessed using the French Disease-Related Groups (DRG) and the national unit cost scale. Ambulatory care, such as visits to a general practitioner or a specialist, medical, paramedical and other acts, laboratory tests, medication, medical equipment and non-medical costs were evaluated according to the appropriate reimbursement tariffs used by the French social health insurance. Costs were expressed in Euros, according to costings for 2012. The education program was evaluated at a rate of €250 per patient per year, according to the flat rate assigned to education programs in public and private hospitals in France (Ministère de la santé, 2013). This flat rate did not include the costs of the PD education nurses’ salaries, which are funded by the French hospitals.

Cost-effectiveness analyses compared costs and outcomes (changes in UPDRS II and III scores reflecting specific disability related to PD) for the two groups of patients. The incremental cost-effectiveness ratio (ICER) was calculated as follows: difference in mean annual total cost between the TTBI and no TTBI groups / difference in UPDRS II and III changes over one year between the TTBI and no TTBI groups. Cost and effectiveness discounting was not calculated because of the short follow-up period [18]. To account for the skewed normality of the costs and effectiveness data, probabilistic sensitivity analyses were performed using a bootstrap resampling (1000 replications) on the cost and effectiveness pairs.

### Statistical analysis

The sample size calculation was based on PDQ-39 values in PD patients [19]. For the detection of a 10-point difference at the 5% significance level, with 80% power and a standard deviation of 19, 57 patients were needed in each group. Assuming that some of the patients were likely to withdraw from the study, 60 patients were included in each group.

Qualitative variables were compared between groups, using the χ^2^-test (or Fisher’s exact test if the expected numbers per cell were small). Student’s *t*-test was used to compare the distribution of quantitative data (or the Mann-Whitney U test if the distribution departed from normality or if the assumption of homoscedasticity was rejected).

Covariance analysis (ANCOVA) with disease duration as a covariable for the adjusted analysis was used to compare changes to the QOL scores and quantitative clinical parameters. To evaluate the effect of the education program on QOL, changes in each dimension of QOL between the baseline and the visit after 12 months of follow- up were compared, between the groups, using a two-side binomial exact test.

Probabilistic sensitivity analysis was used to construct the 95% confidence intervals around the incremental costs per one-point unit improvement in UPDRS II and III, based on the 2.5^th^ and the 97.5^th^ percentiles. The output of bootstrap resampling was used to determine graphically, the 95% credible region of the ICER using the 95% confidence ellipse method.

All values are expressed as means ± standard deviation. Differences in scores changes are presented with the 95% confidence interval (95% CI). A *P*-value ≤ 0.05 was significant. Missing clinical data at 12 months of follow up were imputed by the LOCF method and an intention-to-treat analysis was used. Data were analyzed using SAS^®^ 9.2 and STATA^®^ 12 statistical software®.

## Results

### Clinical outcomes

**Fig 1:**
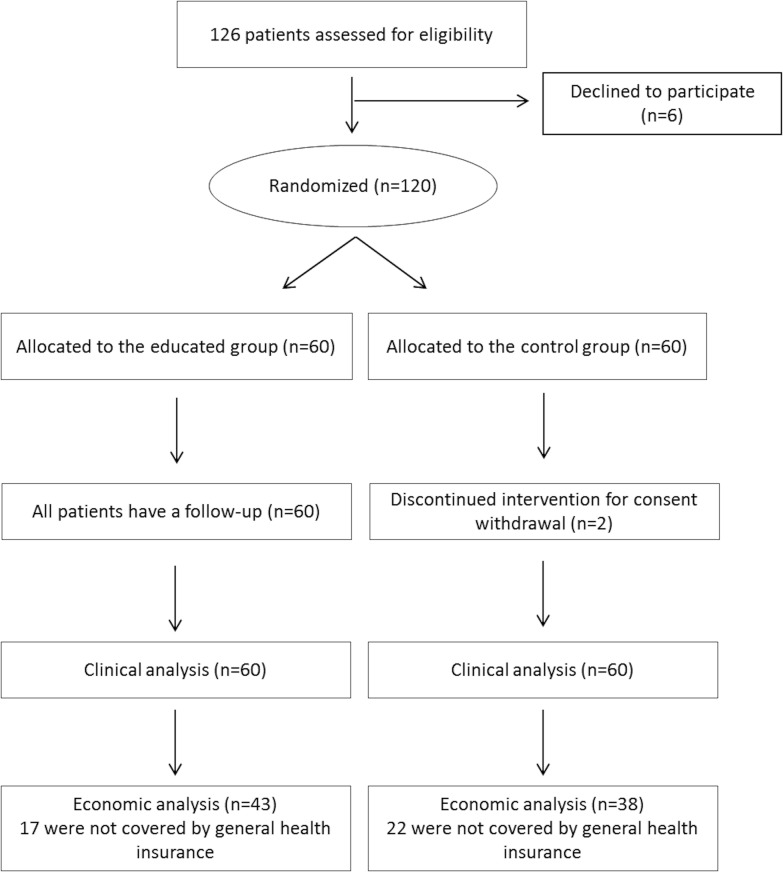
Flow chart of participants in the study.

A total of 120 PD patients were randomized to TTBI (*n* = 60) and no TTBI (*n* = 60) groups. Recruitment was from December 2008 to April 2010 and the last follow-up was in April 2011. All but two of the patients completed the study. Both were from the control group and withdrew their consent (Fig 1).

The demographic and clinical characteristics of the patients at baseline are reported in Table 1, except SF36 scores available in the supporting files (S1 Table). No significant difference was observed between the groups in terms of age, sex, disease severity, marital status or educational background. Nevertheless, disease duration was shorter in TTBI group than in no TTBI group (4.9±4.3 vs. 6.8±5.2 *P* = 0.03, respectively).

**Table 1:** Demographics and clinical characteristics of the TTBI and no TTBI groups at baseline.

	TTBI (n = 60)	no TTBI (n = 60)
Male/female	40/20	31/29
Age (years)	62·1±7·1	65·1±9·2
Years since diagnosis	4·9±4·3	6·8±5·2*
Number of chronic conditions	1·9±1·2	2·0±1·4
Education level:		
Left school before age of 18 years	23	31
Educated until age of18 years	9	8
Higher education	28	20
Employed/unemployed	13/47	9/51
With a partner/single	49/11	44/16
Hoehn & Yahr stage		
Stage 1/stage 2/stage 3	12/36/13	11/35/12
UPDRS score		
Score I (mental, behavioral, and mood state)	1·1±1·4	1·2±1·6
Score II (daily life activities)	6·8±4·2	7·9±4·8
Score III (motor evaluation)	12·2±7·2	14·3±1·9
Score IV (treatment complications)	1·3±2·0	1·7±2·4
Total score	21·4±11·3	25·2±13·9
PDQ39		
Mobility	22·0±19·6	27·4±21·4
Activities of daily living	23·8±17·4	27·9±15·4
Emotional well-being	31·8±21·1	34·4±18·5
Stigma	25·9±19·3	29·7±19·7
Social support	10·0±18·9	10·9±15·4
Cognition	28·3±18·4	34·0±17·7
Communication	18·8±18·5	23·9±18·7
Bodily discomfort	38·5±20·2	43·9±23·2
Anxiety and Depression		
Anxiety	8·8±4·6	8·8±3·6
Depression	5·6±3·3	6·9±3·6*
Total score	14·4±7·1	15·7±6·2

Values are means±SD

*: *P*<0.05

At baseline, QOL, motor and psychological states did not differ significantly between the groups, except for the HADS depression subscale, which was lower in the educated group, (*P* = 0.04; Table 1).

At 12 months, although there was a trend towards improvement of quality of life in the TTBI group compared to the no-TTBI, the PDQ-39 changes between the groups were not significantly different for each PDQ-39 dimension (Table 2).

**Table 2:** Comparison of the changes (between the baseline and at 12 months) of quality of life, motor and psychological scale scores for both groups.

	TTBI (*n* = 60)	no TTBI (*n* = 60)	*P* (non adjusted)	*P* (adjusted)	Difference of no TTBI vs TTBI (95% CI)
PDQ-39					
Mobility	0.66±15.15	3.89±13.4	0.24	0.32	3.21(-2.12; 8.56)
ADL	-1.66±13.08	1.56±15.73	0.24	0.37	3.22 (-2.21; 8.65)
Emotional well being	-4.37±16.52	-0.07±13.93	0.14	0.19	4.30 (-1.38; 9.99)
Stigma	-6.68±17.37	-4.59±17.03	0.50	0.60	2.09 (-4.16; 8.33
Social Support	-2.03±13.5	0±15.85	0.40	0.43	2.03 (-3.36; 7.42)
Cognition	-2.56±14.74	2.13±13.88	0.07	0.11	4.69 (-0.51; 9.88)
Communication	-2.73±15.02	0.01±15.34	0.32	0.42	2.74 (-2.80; 8.23)
Bodily discomfort	-2.78±15.77	0.98±14.64	0.18	0.33	3.76 (-1.75; 9.26)
UPDRS					
UPDRS I	-0.40±1.32	0·44±1·67	<0.01	0.01	0.84 (0.29; 1.39)
UPDRS II	-0.95±3.24	1.37±4.15	<0·001	<0.01	2.32 (0.97; 3.67)
UPDRS III	-0.48±5.54	1.00±6.23	0.17	0·26	1.48 (-0.65; 3.62)
UPDRS IV	0.17±1.46	0.63±1.41	0·08	0·05	0.46 (-0.07; 0.98)
Total score	-1.69±8.04	3.46±9.53	<0.01	<0.01	5.15 (1.94; 8.37)
Anxiety and depression					
Anxiety	-1.00±3.28	0.08±2.72	0·05	0·08	1.08 (-0.01; 2.18)
Depression	-0.35±2.58	-0.22±3·1	0·80	0·90	0.13 (-0.91; 1.17)
Total score	-1.35±4.7	-0.14±4.78	0·16	0·24	1.21 (-0.51; 2.94)

Values are means±SD; *P* is adjusted on disease duration

**Fig 2:**
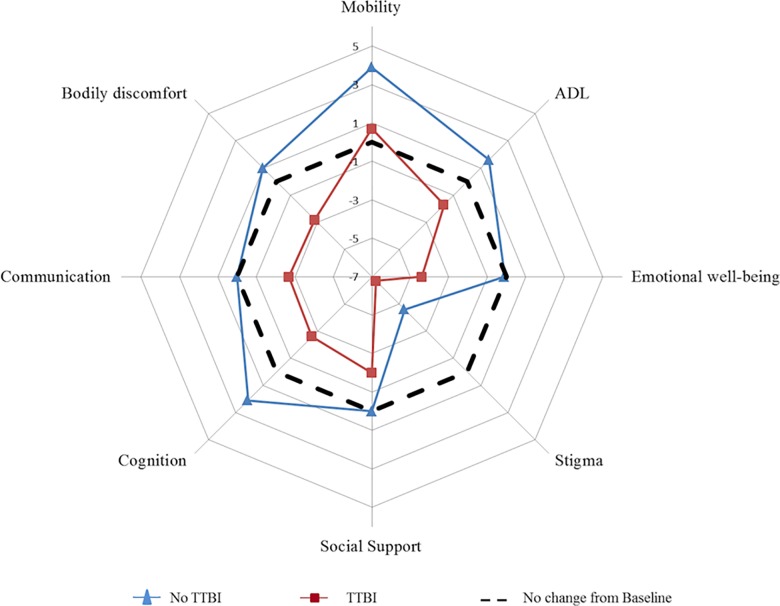
Evaluation of the change in PDQ-39 score between the baseline and at 12 months in the two groups of PD patients. The spider chart shows the difference in each PDQ-39 dimension between the baseline and at 12 months. The dotted line indicates an absence of change in the PDQ-39 score over the 12-month period. Quality of life improved when the point plotted is below the dotted line and worsened when it is above the dotted line.

Over one year, each dimension of PDQ39 (8/8) exhibited better changes in the TTBI group vs no-TTBI group (binomial exact test, *P* = 0·008; Table 2). In addition, improvements were observed for a larger number of PDQ39 dimensions (improvement in seven dimensions vs. worsening in one dimension) in the TTBI group than in no -TTBI group (improvement in one dimension vs. worsening in seven dimensions) (Fig 2).

At 12 months, SF-36 changes were not significantly different between the groups except for the social functioning dimension which was significantly improved in the TTBI group (*P* = 0.01; S2 Table, supporting file).

Over one year, 6 dimensions of SF36 (6/8) exhibited better changes in the TTBI group vs no-TTBI group (binomial exact test, *P* = 0·2; S2 Table, supporting file). In addition, improvements were observed in 4/8 of SF36 dimensions in the TTBI group (improvement in 4 dimensions vs. worsening in 4 dimensions) in the TTBI group and in no -TTBI group (improvement in one dimension vs. worsening in seven dimensions) (S1 Fig, supporting file).

A significant difference was observed in, part I, part II and total UPDRS scores after 12 months between the groups even after adjustment on disease duration (*P*≤0.01), except for part III and part IV (Table 2).

At 12 months, although there was a trend towards improvement of anxiety in the TTBI group compared to the no-TTBI group, comparison of the HADS scores between the groups was not significantly different (Table 2)

All data at six months are presented in supporting files (S3 Table). PDQ39 and SF-36 changes were not significantly different between the groups except for the physical functioning dimension of SF-36 which significantly improved in the TTBI group (*P* = 0.04). There was a significant difference in the UPDRS scores between the groups (*P*≤0.01) except for part IV but no significant difference was found in the HADS scores.

After one year, the mean patient satisfaction score was 8·8±1·1 and 53 of 60 patients from the TTBI group wished to continue the education program.

### Economic evaluation (Table 3)

Only 81 of the 120 patients in the sample (43 in the educated group and 38 in the control group) were included in the French social health insurance database (Fig 1). Characteristics of these patients are summarized in Table 3.

**Table 3:** Economic analysis: patient characteristics and costs.

		TTBI (*n* = 43)	no TTBI (*n* = 38)	*p*
**Patient characteristics**				
Age (years)		62·57 ±6·38	64·72±9·45	0·23
Male/female		28/15	15/23	0·02
Disease duration (years)		7.15±4·48	7·74±4·80	0·61
Number of chronic conditions		1·49± 1.26	2.11±1.59	0·08
UPDRS II changes		-1.21±3.16	1.18±4.03	<0.01
UPDRS III changes		-0.84±5.09	2.00±6.80	0.01
**Mean annual costs per patient (Euros)**							
**Hospitalization**		**1715±5,651**	**1550±2380**	0·34
**Ambulatory care**		**1539±1,830**	**1746±1790**	0·44
Medical visits		350±176	374±192	0·74
Medical acts^a^		143±147	189±145	0·08
Laboratory tests		58±56	104±114	0·05
Paramedical acts^b^		792±1,734	934±1,674	0·72
Others^c^		196±420	146±202	0·23
**Treatments**		**3693±5,164**	**3924±6076**	0·68
Drugs		3.064±5,164	3296±2847	0·67
*Antiparkinsonian drugs*		*2427±2*,*009*	*2590±2576*	*0·95*
*Psychotropic drug*^*d*^		*76±137*	*145±236*	*0·12*
*Other drugs*		*563±540*	*570±643*	*0·69*
Medical equipment^e^		629±3,511	627±3529	0·36
**Transportation**		**126±448**	**227±375**	0·07
**Education program**		**250**	**-**	-
**Total costs**		**7323±8560**	**7372±8022**	0·50
**Incremental cost-effectiveness ratios **				
	Incremental cost (€)	Incremental effectiveness	Observed ICER^f^	Simulated boostrap ICER (95%)CI^g^
UPDRS II score	-49	-2.39	-21	9 (-1,738; + 2,515)
UPDRS III score	-49	-2.84	-17	-19 (-1,898; + 2,506)

Values are means±SD

^a^: Diagnostic and therapeutic acts

^b^: performed by a nurse, physiotherapist, orthoptist or speech therapist

^c^: dental acts, spa therapy

^d^: antipsychotic drugs, antidepressants, benzodiazepines, non-benzodiazepine hypnotic drugs

^e^: respiratory assistance, apomorphine pump, orthosis

^f^: ICER is expressed as € per one point improvement in UPDRS II & III

^g^: results of non-parametric boostrap ICER is presented as median and 95%. CI

Mean annual costs did not differ significantly between the groups, at €7323±8560 for the TTBI group and €7372±8022 for the no-TTBI group. The main cost was treatment, in both groups, accounting for 52% of total costs in the TTBI group compared to 53% in the no-TTBI group (*P* = 0.97). Antiparkinsonian drugs accounted for 79% of medication costs in both groups.

Hospitalization and ambulatory care costs did not differ significantly between the groups. Nevertheless, among the costs of ambulatory care, there was a trend towards lower costs for laboratory testing (*P* = 0.05) and medical acts (*P* = 0·08) in the TTBI group. In addition, transportation costs tend to be lower in the TTBI group (*P* = 0.07).

Cost-effectiveness results are summarized in Table 3. A negative ICER of €21per score point gained on the UPDRS II scale and €17 per score point gained on the UPDRS III scale were calculated. The results of the non-parametric bootstrapping with 1000 replications showed a median positive ICER of €9 per unit improvement in the UPDRS II score with a CI-95% ranging from €-1738 to €2515 and a median negative ICER of €19 per unit improvement in the UPDRS III score with a CI-95% ranging from €-1898 to €2506.

**Fig 3:**
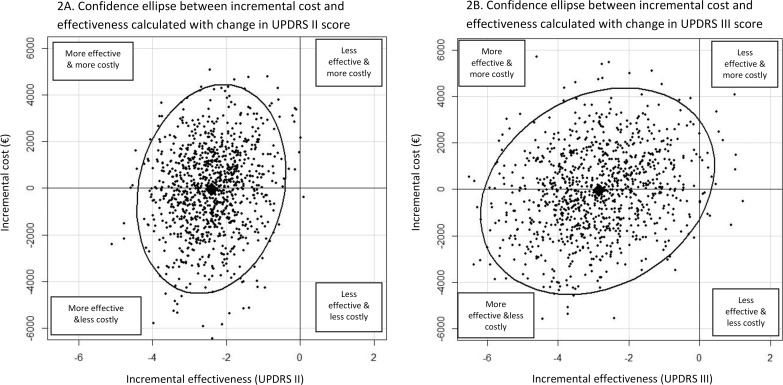
95% credible region of the ICER using the confidence ellipse method. These figures show the cost-effectiveness plane for therapeutic education in PD compared to conventional care based on 1000 bootstrap estimates of the difference in cost and effectiveness. Incremental effectiveness and incremental cost are plotted on the horizontal and vertical axes, respectively. Each dot represents the ICER for one simulation. Lozenges represent the observed ICER. Ellipses represent a 95% confidence region around the ICER for the therapeutic education strategy compared to conventional care. Most of the dots are found in the left areas, showing that therapeutic education is cost-effective (more effective and more costly) or cost saving (more effective and less costly).

In Addition, Fig 3 shows a 95% credible region of the ICER represented by confidence ellipses performed on the simulated bootstrap costs and effectiveness. Most of the simulated ICERs are found either in the cost-effective or the cost-saving area.

## Discussion

This study was the first of this kind to evaluate the effectiveness and cost of an education program for PD in France. The program improved QOL and motor status without increasing the annual total medical cost per patient and therefore could be considered as a cost saving intervention.

For neurological disorders, such as multiple sclerosis, education programs have already been shown to improve patient behavior with respect to health status [20]. For PD, only a few studies have evaluated education programs, with various methodologies. Two studies compared QOL before and after a standardized education program in seven European countries, but they found no significant change in QOL, particularly when assessed withPDQ39 [5; 9]. Conversely, two randomized controlled trials assessing two different education programs reported a significant improvement in the global PDQ39 score in the educated group compared to control group [12–13]. This study showed no significant effect of the education program on PDQ39 changes at 12 months. However, the effect sizes of an education program on each PDQ39 dimension are very close to those defined as the minimally important difference (subjectively meaningful to patients) [21]. Thus, the observed differences in QOL between the groups are clinical relevant, but not statistical significant. Study of a larger number of patients may reveal statistical significance. Improved changes in each dimension in the TTBI group compared to the no-TTBI group also suggested that our education program has a positive effect on QOL in PD patients.

The education program induced significant improvement in only one dimension of the SF36 questionnaire. SF36appears not to be the best tool to assess the QOL in PD because SF36 is too general and, not sensitive enough to take into account the specific issues relating to PD patients. The findings of this study are consistent with those of Lindskov *et al*. [22], who reported no improvement in QOL assessed with the short version of SF36 (SF12 scale).

Improvement observed in the UPDRS subscales and total score supported the education program. The UPDRS, which is classically used to assess the effect of drugs on PD symptoms, may also be considered a good indicator of the impact of this kind of intervention. Our results are consistent with those of Guo *et al*. [12], who also reported an improvement in UPDRS II and III scores for PD patients following an education program.

Regarding psychological status, the study duration was probably too short to adequately reflect an effect of the education program. Nevertheless, most of the studies assessing mood status have reported no change in the depression score of the Self-Rating Depression Scale (SDS) [6; 9–10; 13]. Although it was not assessed, adherence to therapy could be one of the reasons for QOL improvement because it is an expected consequence of an education program.

These results suggest that our education program could have a positive effect on symptom outcomes and on the perceived health of PD patients. This conclusion is also supported by the positive evaluations of the program received from the participants. Based on both the satisfaction score obtained and the number of patients wishing to continue this program, most patients found it beneficial. Patients consistently reported that they had found the individual sessions to develop and hone the necessary skills in their daily life, and the exchange of experiences with other patients during group sessions very useful.

The annual direct costs per patient amounted to €7372 for the no-TTBI group and €7323 for the TTBI group, which is consistent with the mean annual direct costs ranging from €3360 to €8160 and the semi-annual costs ranging from €1760 to €6040 reported for European countries, [23–24]. A large SD of the mean annual overall medical costs was found probably for two reasons: firstly as it was a randomized controlled trial, all cost and not specific costs due to PD were assessed during a one year period and secondly, costs are never normally distributed. In this study, treatment cost was the main expenditure similar to the findings of previous publications in six European countries (except France) which reported a semi-annual cost of antiparkinsonian drugs ranging from €490 to €2960 [24]. In France, annual cost of medication is €1022 but this study was conducted in 1999 and new medication has resulted in higher costs today [25].

The education program for PD patients did not increase the annual direct costs, even when taking into account the cost of the education program itself. Also it did not generate extra costs due to additional paramedical acts or transportation.

Moreover, laboratory and medical act costs seemed to decrease in the educated group. This may be because therapeutic education improves the patients’ understanding of their disease, increasing well-being and decreasing the need for additional examinations.

The cost-effectiveness of an education program for PD was evaluated to shed light on the medical and economic consequences of this new non-pharmacologic approach.

Regarding the observed ICERs, this study found that the education program dominated usual care since it was less costly and more effective in the reduction of UPDRS II and III. The ICER required to achieve an additional one-point improvement allowed a saving of €21 per patient for UPDRS II and € 17 per patient for UPDRS III. The simulated ICER confirmed these results and even the values of the upper range of the confidence interval remained lower than the ICER values reported in Deep Brain Stimulation (€6729) [26].

The study had several limitations. The first limitation was the low sample size, which consequently underpowered the trial. The second limitation was that the sex ratio differed significantly between the two groups as only 81 patients were included in the economic analysis; therefore our findings are subject to attrition bias. Moreover, this loss of data undoubtedly decreased the overall statistical power for detecting differences in cost outcomes. Another limitation of this analysis was that it considered only direct costs, it did not include indirect and informal costs, such as lost productivity, and thus probably underestimated costs from a societal perspective. The study was based on a one year follow-up period, which could be considered too short. A longer follow-up duration might have revealed larger changes in QOL and psychological status and greater savings in terms of medical costs. The existence of a significant difference in the depression score between the groups at baseline despite the randomization process makes interpretation of the education program effect on the depression status difficult. Finally, lack of function utility associated with the specific PDQ39 questionnaire did not allow cost utility analysis.

## Conclusion

Therapeutic education is a component of prevention strategies and might be considered as a cost-saving intervention for a French health insurance payer in PD. Money spent today will provide medical aid some years later. This cost effectiveness study should be considered as a pilot study, paving the way for additional studies with larger numbers of patients and careers and a longer follow-up period.

## Supporting Information

S1 CONSORT Checklist (DOC)

S1 Dataset (XLSX)

S1 Fig Evaluation of the change in SF36 score between baseline and 12 months in the two groups of PD patients. The spider chart shows the difference in each SF36 dimension between baseline and 12 months. The dotted line indicates an absence of change in SF36score over the 12-month period. Quality of life has improved when the point plotted is above the dotted line and worsened when it is below the dotted line.
(TIF)

S1 Protocol Study protocol, French version. (DOC)

S2 Protocol Study protocol, English version. (DOC)

S1 Table Baseline status of SF36 scale of the TTBI and no TTBI groups. (DOCX)

S2 Table Comparison of the changes (between the 12 months and baseline) of quality of life (SF 36) in the 2 groups. (DOCX)

S3 Table Comparison of the changes (between the 6 months and baseline) of quality of life UPDRS and psychological scores. (DOCX)

## References

[1] Von Campenhausen S, Bornschein B, Wick R, Bötzel K, Sampaio C, Poewe W, et al Prevalence and incidence of Parkinson's disease in Europe. Eur Neuropsychopharmacol. 2005 8;15(4):473–90. Review. 10.1016/j.euroneuro.2005.04.007 15963700

[2] Schrag A, Jahanshahi M, Quinn N. How does Parkinson's disease affect quality of life? A comparison with quality of life in the general population. MovDisord. 2000;15:1112–8. 10.1002/1531-8257(200011)15:6<1112::aid-mds1008>3.0.co;2-a 11104193

[3] Terriff DL, Williams JV, Patten SB, Lavorato DH, Bulloch AG. Patterns of disability, care needs, and quality of life of people with Parkinson's disease in a general population sample. Parkinsonism Relat Disord. 2012; 18: 828–32. 10.1016/j.parkreldis.2012.03.026 22542396

[4] Rajendran PR, Thompson RE, Reich SG. The use of alternative therapies by patients with Parkinson’s disease. Neurology 2001; 57: 790–794. 10.1212/wnl.57.5.790 11552005

[5] Smith Pasqualini MC; Members of the EduPark consortium. An innovative education programme for people with Parkinson's disease and their carers. Parkinsonism Relat Disord. 2006; 12: 478–85. 10.1016/j.parkreldis.2006.05.003 16781881

[6] Shimbo T, Goto M, Morimoto T, Hira K, Takemura M, Matsui K, et al Association between patient education and health-related quality of life in patients with Parkinson's disease. Qual Life Res. 2004;13: 81–9. 10.1023/b:qure.0000015306.59840.95 15058790

[7] Montgomery EB Jr, Lieberman A, Singh G, Fries JF. Patient education and health promotion can be effective in Parkinson's disease: a randomized controlled trial. PROPATH Advisory Board. Am J Med.1994; 97: 429–35. 10.1016/0002-9343(94)90322-0 7977431

[8] Mercer BS. A randomized study of the efficacy of the PROPATH Program for patients with Parkinson disease. Arch Neurol. 1996; 53: 881–4. 10.1001/archneur.1996.00550090087014 8815853

[9] Macht M, Gerlich C, Ellgring H, Schradi M, Rusiñol AB, Crespo M, et al Patient education in Parkinson's disease: Formative evaluation of a standardized programme in seven European countries. Patient Educ Couns. 2007; 65: 245–52. 10.1016/j.pec.2006.08.005 16965885

[10] Grosset KA, Grosset DG. Effect of educational intervention on medication timing in Parkinson's disease: a randomized controlled trial. BMC Neurol. 2007 16; 7: 20 10.1186/1471-2377-7-20 17634109 PMC1931606

[11] Lindskov S, Westergren A, Hagell P. A controlled trial of an educational programme for people with Parkinson's disease. J Clin Nurs. 2007;16: 368–76. 10.1111/j.1365-2702.2007.02076.x 17931329

[12] Guo L, Jiang Y, Yatsuya H, Yoshida Y, Sakamoto J. Group education with personal rehabilitation for idiopathic Parkinson's disease. Can J Neurol Sci. 2009; 36: 51–9. 10.1017/s0317167100006314 19294889

[13] A’Campo LEI, Spliethoff-Kamminga NGA, Macht M, The EduPark Consortium, Roos RAC. Caregiver education in Parkinson’s disease: formative evaluation of a standardized program in seven European countries. Qual Life Res 2010; 19: 55–64. 10.1007/s11136-009-9559-y 19946755 PMC2804793

[14] Andlin-Sobocki P, Jönsson B, Wittchen HU, Olesen J. Cost of disorders of the brain in Europe. Eur J Neurol. 2005; 12 Suppl 1: 1–27. 10.1111/j.1468-1331.2005.01202.x 15877774

[15] Lindgren P, von Campenhausen S, Spottke E, Siebert U, Dodel R. Cost of Parkinson's disease in Europe. Eur J Neurol. 2005; 12 Suppl 1: 68–73. 10.1111/j.1468-1331.2005.01197.x 15877783

[16] Ory Magne F, Arcari C, Canivet C, Sarrail M, Fabre MH, Mohara C, et al A therapeutic educational program in Parkinson’s disease: ETPARK, Rev Neurol (Paris). 2014; 170: 128–33 10.1016/j.neurol.2013.08.007 24267951

[17] Jenkinson C, Fitzpatrick R, Peto V, Greenhall R, Hyman N., The Parkinson's Disease Questionnaire (PDQ-39): development and validation of a Parkinson's disease summary index score. Age Ageing. 1997; 26: 353–7. 10.1093/ageing/26.5.353 9351479

[18] Drummond MF, Sculpher MJ, Torrance GW, O’Brien JB, Stoddart GL. Methods for the economic evaluation of health care programmes. 3rd ed. Oxford, Oxford University Press; 2005.

[19] Brefel-Courbon C, Desboeuf K, Thalamas C, Galitzky M, Senard JM, Rascol O, et al Clinical and economic analysis of spa therapy in Parkinson's disease. Mov Disord. 2003; 18: 578–84. 10.1002/mds.10404 12722173

[20] Demaille-Wlodyka S, Donze C, Givron P, Gallien P; ETP Sofmer Group. Self care programs and multiple sclerosis: physical therapeutics treatment—literature review. 2011; 54: 109–28. 10.1016/j.rehab.2011.01.003 21388907

[21] Peto V, Jenkinson C, Fitzpatrick R. Determining minimally important differences for the PDQ-39 Parkinson's disease questionnaire. Age Ageing. 2001;30: 299–302. 10.1093/ageing/30.4.299 11509307

[22] Lindskov S, Westergren A, Hagell P. A controlled trial of an educational programme for people with Parkinson's disease. J Clin Nurs. 2007;16: 368–76. 10.1111/j.1365-2702.2007.02076.x 17931329

[23] Lindgren P, von Campenhausen S, Spottke E, Siebert U, Dodel R. Cost of Parkinson's disease in Europe. Eur J Neurol. 2005 6;12 Suppl 1: 68–73. 10.1111/j.1468-1331.2005.01197.x 15877783

[24] Von Campenhausen S, Winter Y, Rodrigues e Silva A, Sampaio C, Ruzicka E, Barone P, et al Costs of illness and care in Parkinson's disease: an evaluation in six countries. Eur Neuropsychopharmacol. 2011; 21:180–91. 10.1016/j.euroneuro.2010.08.002 20888737

[25] LePen C1, Wait S, Moutard-Martin F, Dujardin M, Ziégler M. Cost of illness and disease severity in a cohort of French patients with Parkinson's disease. Pharmacoeconomics. 1999; 16:59–69. 10.2165/00019053-199916010-00006 10539122

[26] Dams J, Siebert U, Bornschein B, Volkmann J, Deuschl G, Oertel WH, et al Cost-effectiveness of deep brain stimulation in patients with Parkinson's disease. Mov Disord. 2013; 28: 763–71. 10.1002/mds.25407 23576266

