# Supplementary figures and images for: Clinical Impact and Cost-Effectiveness of an Education Program for PD Patients: A Randomized Controlled Trial
Source: PLoS One. 2016 Sep 29;11(9):e0162646. doi: 10.1371/journal.pone.0162646 (PMC5042480; doi:10.1371/journal.pone.0162646)

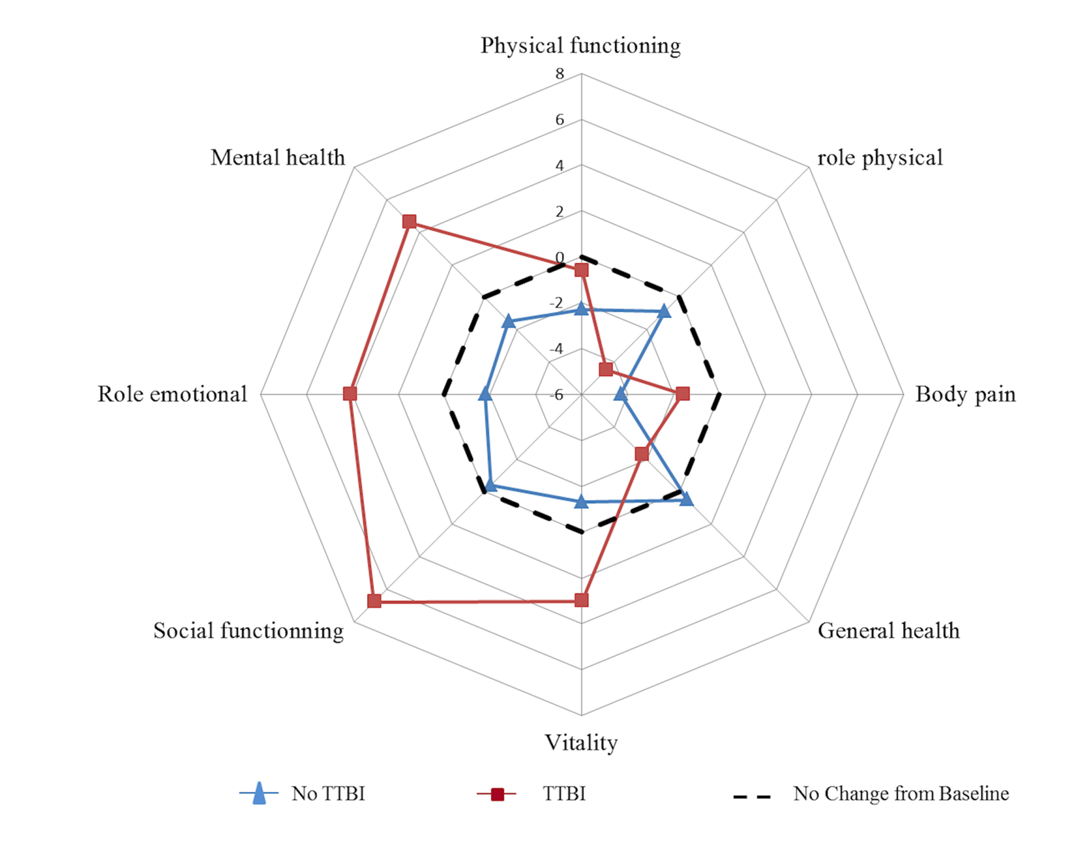

Supplement: S1 Fig — The spider chart shows the difference in each SF36 dimension between baseline and 12 months. The dotted line indicates an absence of change in SF36score over the 12-month period. Quality of life has improved when the point plotted is above the dotted line and worsened when it is below the dotted line. (TIF) [file pone.0162646.s003.tif]
