# Supplementary material for: Clinical Impact and Cost-Effectiveness of an Education Program for PD Patients: A Randomized Controlled Trial
Source: PLoS One. 2016 Sep 29;11(9):e0162646. doi: 10.1371/journal.pone.0162646 (PMC5042480; doi:10.1371/journal.pone.0162646)
Supplement: S1 Protocol — (DOC) [file pone.0162646.s004.doc]

| **TITRE** : | **EVALUATION D’UN PROGRAMME D’EDUCATION THERAPEUTIQUE DANS LA MALADIE DE PARKINSON.**  **Etude ETPARK** | |
| --- | --- | --- |
| **ORGANISME** : | Nom,  Adresse, Téléphone : | CHU de TOULOUSE  Hôtel Dieu, 2 rue Viguerie, TSA 80035  31 059 Toulouse Cedex 9  Contact : Llau Marie-Elise  Tel : 05 61 77 87 71 |
| **RESPONSABLE DE L’ETUDE** : | Nom,  Adresse, Téléphone : | Dr Christine Brefel-Courbon  Service de Neurologie, Pole Neuroscience,  CHU Toulouse  Tel : 05 61 14 75 16  Email : brefel@cict.fr |
| **MEDECINS ASSOCIES**: | Nom,  Adresse : | Dr Fabienne Ory, Service de Neurologie,  Pole Neuroscience, CHU Toulouse  Dr Nelly Fabre, Service de Neurologie,  Pole Neuroscience, CHU Toulouse  Pr. Olivier Rascol, Service de Neurologie,  Pole Neuroscience, CHU Toulouse  Pr. Michel Clanet, Coordonateur du Pôle Neuroscience, CHU Toulouse  Tel : 05 61 14 75 16 |
| **AUTRES PARTICIPANTS :** | Nom,  Adresse : | Pr. Claire Thalamas (soutien logistique et méthodologique)  Centre d’Investigation Clinique, CHU Toulouse  Tel : 05 61 77 91 03  Dr Gérard TAP- biostatisticien,  Centre d’Investigation Clinique, CHU Toulouse  Céline Arcari, IDE,  Pole Neuroscience CHU Toulouse  Dr Laurent Molinier (Département d'Information Médicale du CHU de Toulouse)  Dr Robert Bourrel (CPAM) |
| **TYPE D’ETUDE** : | *Etude randomisée, en ouvert* | |
| **DUREE DU PROJET** : | 3 ans | |
| **NOMBRE DE SUJETS** : | 120 patients | |
| **LOI DE SANTE PUBLIQUE** du 9 août 2004 | OUI, recherche visant à évaluer des soins courants | |
| **N° enregistrement**: | 2008-A00720-55 | |
| **MOTS CLES** : | Parkinson, éducation thérapeutique, qualité de vie, étude médico-économique | |

1. RESUME

**CONTEXTE DU PROJET DE RECHERCHE :**

Les traitements antiparkinsoniens ne sont que symptomatiques et souvent insuffisants surtout lorsque la maladie est évolutive. Un programme d’éducation thérapeutique (ETP), en complément du traitement médicamenteux antiparkinsonien habituel, pourrait exercer une action bénéfique sur les différentes composantes de la maladie et donc améliorer la qualité de vie du Parkinsonien.

Il existe à ce jour quelques publications faisant état de l’intérêt de l’éducation thérapeutique dans la maladie de Parkinson qui rapportent une amélioration de certains aspects de la qualité de vie, de l’état psychologique et de l’observance médicamenteuse des patients Parkinsoniens. A notre connaissance aucune étude de ce type n’a été réalisée en France.

**OBJECTIFS DU PROJET DE RECHERCHE** :

L’objectif principal de l’étude est de montrer l’amélioration de la qualité de vie de patients parkinsoniens bénéficiant d’un programme d’ETP par rapport à des patients parkinsoniens n’en bénéficiant pas. Les objectifs secondaires de l’étude sont de comparer l’état moteur, l’état psychique, l’adaptation sociale et les coûts médicaux, chez les deux groupes de patients parkinsoniens.

**METHODES ET OUTILS :**

Il s’agit d’une étude monocentrique, comparative prospective, randomisée, ouverte entre 2 groupes de patients parkinsoniens (le premier bénéficiant d’un programme d’ETP pendant 12 mois, le second ne bénéficiant de ce programme). Nous évaluerons la qualité de vie des Parkinsoniens grâce à une échelle spécifique (PDQ39) et une échelle généraliste (SF-36) de qualité de vie à 6 et 12 mois chez 120 Parkinsoniens (60 dans chaque groupe). Nous évaluerons aussi l’état moteur, l’état psychologique, l’adaptation sociale des deux groupes de patients. Nous recueillerons les coûts médicaux grâce à un carnet de consommation de soins spécifiant médicaments, hospitalisations, consultations…

**DEROULEMENT ET DUREE DU PROJET** :

Au sein de chaque groupe, les différents paramètres seront recueillis en baseline puis à 6 et 12 mois au cours d’une consultation habituelle de suivi en Neurologie. Le programme d’ETP comprendra une consultation initiale puis des consultations de suivi individuel (1 à 3 par trimestre), une séance thématique en groupe (2 à 3 par semestre) puis une consultation d’évaluation au bout de 12 mois. La durée d’inclusion sera de 24 mois et la durée totale du projet de 36 mois.

**IMPACT DES RESULTATS ATTENDUS :**

Les résultats escomptés sont une amélioration de la qualité de vie des patients parkinsoniens bénéficiant d’un programme d’ETP par rapport à des patients parkinsoniens n’en bénéficiant pas.

Nous faisons l’hypothèse d’un différentiel de coût en faveur des patients parkinsoniens bénéficiant d’un programme d’ETP.

1. JUSTIFICATION SCIENTIQUE ET DESCRIPTION GENERALE

La maladie de Parkinson, avec une prévalence de 150/100 000 habitants, représente la deuxième cause d'affection neuro-dégénérative, derrière la maladie d'Alzheimer (Petit et al, 1994). Elle concernerait environ 2/3 à 3/4 des syndromes parkinsoniens et toucherait à peu près 1,5 % des sujets âgés de plus de 60 ans (Tison, 1998). Maladie neurologique chronique et dégénérative, elle se caractérise non seulement par une symptomatologie motrice (akinésie, rigidité, tremblement de repos, anomalies de posture) mais également des troubles cognitifs et psychiques, des perturbations du sommeil, des douleurs, des dysfonctions sexuelles (Fitzsimmons et al, 1993). Cette symptomatologie variée peut donc affecter certains aspects de la vie quotidienne. Plusieurs études ont démontré qu’il existait une altération de la qualité de vie dans la maladie de Parkinson (Karlsen et al, 1999 ; Schrag et al, 2000 ; Riazi et al, 2003). Les traitements antiparkinsoniens ne sont que symptomatiques et souvent insuffisants surtout lorsque la maladie est évolutive. Près de la moitié des patients Parkinsoniens ont recours à au moins une thérapie alternative (Rajendran et al, 2001).

Un programme d’éducation thérapeutique, en complément du traitement médicamenteux antiparkinsonien habituel, pourrait exercer une action bénéfique sur les différentes composantes de la maladie et donc améliorer la qualité de vie du Parkinsonien.

Il existe à ce jour quelques publications faisant état de l’intérêt de l’éducation thérapeutique dans la maladie de Parkinson (Simons et al, 2006 ; Shimbo et al, 2004 ; Montgomery et al, 1994 ; Mercer et al, 1996 ; Macht et al, 2007, Grosser et al, 2007). Cependant, beaucoup d’entre elles n’évalue pas l’éducation thérapeutique en utilisant une méthodologie satisfaisante (Simons et al, 2006 ; Shimbo et al, 2004 ; Macht et al, 2007). En effet, ces études n’incluent pas de groupe contrôle et démontrent seulement une faisabilité et une satisfaction des patients et des aidants. Trois études comparatives, randomisées effectuées aux états unis et au royaume Uni rapportent une amélioration de certains aspects de la qualité de vie, de l’état psychologique et de l’observance médicamenteuse des patients Parkinsoniens (Montgomery et al, 1994 ; Mercer et al, 1996 ; Grosser et al, 2007). A notre connaissance aucune étude de ce type n’a été réalisée en France.

En France nous pouvons cependant nous inspirer de l’expérience désormais acquise par les diabétologues sur l’intérêt de l’éducation thérapeutique dans la prise en charge du diabète. L’ANAES en 2002 a validé l’éducation thérapeutique comme partie intégrante et nécessaire à la prise en charge des patients diabétiques. Or, le diabète est tout comme la maladie de Parkinson, une affection chronique où l’intervention du patient n’est pas négligeable.

La maladie de Parkinson au quotidien impose en effet, à la personne atteinte (et/ou son accompagnant) :

- de prendre elle-même une partie des décisions qui influenceront l’efficacité de son traitement (alimentation, activité physique, entretien et maîtrise de son corps, auto surveillance symptomatique, hygiène de vie, rendez-vous médicaux et paramédicaux spécialisés adéquats,..)
- et d’assurer la prévention ou le dépistage précoce de possibles complications, notamment iatrogènes.

Notre expérience menée depuis 2005 dans l’unité des mouvements anormaux du CHU de Toulouse montre que l’organisation hebdomadaire d’une réunion des patients hospitalisés atteints la maladie de Parkinson, accompagnés ou non de leur conjoint, sous la responsabilité d’une infirmière et d’un médecin sensibilisés à l’intérêt de l’éducation thérapeutique leur permet de mieux intégrer les principes de leur traitement, les perspectives thérapeutiques à venir, et le pronostic de leur pathologie. Cette expérience pilote débouche sur une meilleure acceptation de leur pathologie, une meilleure compliance au traitement et une facilitation des relations avec les soignants et médecins.

Enfin, le contexte économique actuel rend nécessaire l’évaluation du coût de ce type de prise en charge. Il est possible qu’une amélioration de la qualité de vie et une meilleure gestion de la prise en charge de leur maladie chronique soient responsables d’une baisse de consommation de soins.

Il est donc important de réaliser une étude médicoéconomique afin de déterminer l’impact d’un programme d’éducation thérapeutique dans la maladie de Parkinson.

**Education thérapeutique du patient (ETP)**

Selon l’OMS (1996), l’éducation thérapeutique du patient vise à aider les patients à acquérir ou maintenir les compétences dont ils ont besoin pour gérer au mieux leur vie avec une maladie chronique. Elle fait partie intégrante et de façon permanente de la prise en charge du patient. Elle comprend des activités organisées, y compris un soutien psychosocial, conçues pour rendre les patients conscients et informés de leur maladie, des soins, de l’organisation et des procédures hospitalières et des comportements liés à la sante et à la maladie. Ceci a pour but de les aider (ainsi que leur famille) à comprendre leur maladie et leur traitement, collaborer ensemble et assumer leurs responsabilités dans leur propre prise en charge dans le but de les aider à maintenir et améliorer leur qualité de vie.

Les finalités de l’ETP sont :

- l’acquisition et le maintien par le patient de compétences d’auto soins (adapter les doses de médicaments en fonction de l’état moteur, réaliser des gestes techniques et des soins, mettre en œuvre un programme d’activité physique….) ;

- l’acquisition de compétences d’adaptation psychosociales (savoir gérer ses émotions et gérer son stress vis-à-vis de sa maladie de Parkinson…).

L’ETP s’adresse donc à l’ensemble des patients Parkinsoniens quelque soit le stade de la maladie et à leurs proches :

- Parce qu’elle est complémentaire et indissociable des traitements et des soins, du soulagement des symptômes, de la prévention des complications.

- Parce qu’elle participe à l’amélioration de la santé du patient (biologique, clinique), à l’amélioration de sa qualité de vie et à celle de ses proches.

Ce programme d’ETP est élaboré avec le malade et personnalisé selon ses besoins (personnels ou thérapeutiques). Il facilite ou organise l’intervention d’autres professionnels de santé tels que psychologue, kinésithérapeute, assistante sociale, diététicienne, ergothérapeute ….

Ce programme d’ETP intervient :

- après l’annonce du diagnostic, pour aider à comprendre, reformuler, apporter soutien psychologique ;
- en consultation de suivi régulier du patient, consolidant les compétences, les actualisant pour une meilleure observance, une bonne gestion des effets secondaires et pour optimiser sa qualité de vie ;
- en consultation de suivi approfondi, dans une période difficile, en cas de difficultés d’apprentissage.

Il comprend quatre étapes :

- le diagnostic éducatif, centré sur les besoins du patient (ce qu’il a, ce qu’il fait, ce qu’il sait, ce qu’il croit, qui est-il, quels sont ses projets)
- les compétences à acquérir, objectifs à atteindre pour le patient, objectif thérapeutique, contrat
- les méthodes éducatives, les outils pédagogiques (programme de consultations, participation aux séances collectives)
- l’évaluation régulière de ses progrès: ce que le patient sait, ce qu’il sait faire, ce qu’il a compris, ce qui reste à apprendre, son projet a t’il progressé.

Nous souhaitons donc mettre en place une étude médicoéconomique visant à évaluer l’impact d’un programme d’éducation thérapeutique chez des patients atteints d’une maladie de Parkinson.

1. OBJECTIFS
   1. Objectif principal

L’objectif principal de l’étude est de montrer l’amélioration de la qualité de vie de patients parkinsoniens bénéficiant d’un programme d’éducation thérapeutique par rapport à des patients parkinsoniens n’en bénéficiant pas.

- 1. Objectifs secondaires

Les objectifs secondaires de l’étude sont de comparer l’état moteur, l’état psychique, l’adaptation sociale et les coûts médicaux, chez les deux groupes de patients parkinsoniens.

- 1. Hypothèse de recherche et résultats attendus

Les résultats escomptés sont une amélioration de la qualité de vie des patients parkinsoniens bénéficiant d’un programme d’éducation thérapeutique par rapport à des patients parkinsoniens n’en bénéficiant pas.

Cette amélioration de la qualité de vie pourra être corrélée avec une amélioration de l’état psychologique et/ou de l’état moteur.

Nous faisons l’hypothèse d’un différentiel de coût en faveur des patients parkinsoniens bénéficiant d’un programme d’éducation thérapeutique.

1. METHODOLOGIE
   1. Plan expérimental

Il s’agit d’une étude monocentrique, comparative prospective, randomisée, ouverte entre 2 groupes de patients parkinsoniens (le premier bénéficiant d’un programme d’éducation thérapeutique, le second ne bénéficiant de ce programme),

Les patients parkinsoniens seront répartis par tirage au sort dans le groupe I, (patients bénéficiant du programme d’éducation thérapeutique), et dans le groupe II, (patients ne bénéficiant pas du programme d’éducation thérapeutique).

Dans les 2 groupes, les patients bénéficieront d’une évaluation clinique réalisée avant la randomisation, et après 6 et 12 mois d’inclusion. Cette évaluation prévoit la passation d’auto-questionnaires visant à évaluer la qualité de vie, l’anxiété, la dépression, le retentissement social de ces patients. De plus, lors de chaque visite, les neurologues spécialisés dans la maladie de Parkinson réaliseront un examen clinique, comportant en particulier une évaluation motrice. Enfin, lors de chaque visite, les patients nous remettrons un carnet de bord où seront systématiquement notés chaque consultation médicale (spécialisée ou non), chaque hospitalisation afin de réaliser une évaluation médico économique.

Dans les 2 groupes, les patients bénéficieront d’un suivi régulier et conventionnel par leur neurologue référent.


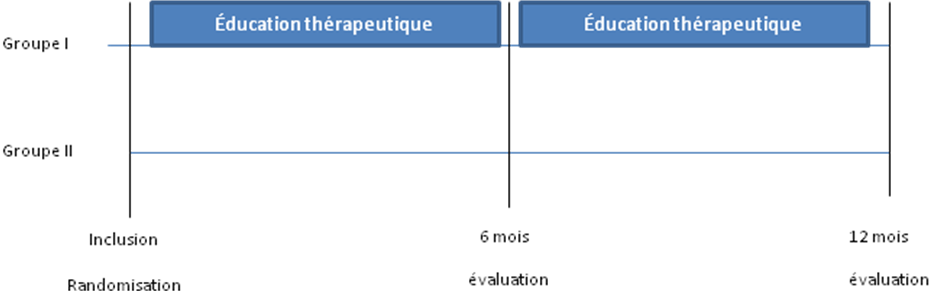


|  | Visite 1 : 1^er^ mois | Visite 2 : 6^ième^ mois | Visite 3 : 12^ième^ mois |
| --- | --- | --- | --- |
| Consentement éclairé | X |  |  |
| Randomisation | X |  |  |
| Auto questionnaires | X | X | X |
| Examen clinique | X | X | X |
| Recueil carnet de bord | X | X | X |

Nous avons choisi de réaliser une étude pragmatique afin de respecter au mieux les conditions de vie habituelles du patient. Nous cherchons donc à interférer au minimum avec la prise en charge habituelle hormis les visites d’ETP.

- 1. Programme d’éducation thérapeutique (ETP)

Ce programme d’Education Thérapeutique a été construit selon les recommandations proposées par la Haute Autorité de Santé dans son guide : Guide Méthodologique-Structuration d’un programme d’éducation thérapeutique du patient dans le champ des maladies chroniques, Haute Autorité de Santé-INPES, Juin 2007 ainsi que selon les recommandations de prise en charge de patients parkinsoniens dans le Guide*-Syndromes Parkinsoniens dégénératifs ou secondaires non réversibles* édité par la HAS. Sur ces bases, notre équipe, composée d’une iDE ayant reçu une formation en éducation thérapeutique (Céline ARCARI) et de neurologues spécialisées dans la MPI (Drs Christine BREFEL COURBON et Fabienne ORY MAGNE), a élaboré un programme d’éduction thérapeutique appliqué aux patients atteints de la MPI.

Ce programme se déroule en 3 étapes qui sont :

### Etape 1 : La Consultation Initiale d’Education

Elle précède toute prise en charge d’éducation. Son contenu sera consigné dans le dossier d’éducation et restera confidentiel. Sa durée est de 45 mn, à adapter selon les possibilités du patient. La Consultation Initiale est réalisée par Céline Arcari et comporte deux étapes :

- L’élaboration du diagnostic éducatif ;
- L’élaboration du contrat d’éducation et du programme du patient.

Le **diagnostic éducatif** est le point de départ de la démarche éducative, c’est la première rencontre entre le patient et le soignant. Il permet au soignant de recueillir des informations précises en appréhendant les différentes dimensions de la vie du patient (données familiales, socioprofessionnelles, connaissance de la maladie, représentations et croyances en matière de santé, ressenti et acceptation de la maladie, projets du patient).

Par ce questionnement, le soignant connaitra mieux le patient, ses ressources personnelles et sociales, ses réactions face à la maladie, sa motivation et les freins à la démarche éducative. De même, il l’amènera à réfléchir sur ses actes et sa perception de la situation, en l’aidant à prendre conscience de ce qu’il sait et ce qu’il ne sait pas.

Pour le patient, le **diagnostic éducatif** initie le processus d’éducation. Il lui permettra de parler de lui, de ses croyances et de ses convictions. Le patient pourra exprimer ses besoins, ses craintes, ses émotions. En parlant de son vécu, il décrira son expérience de la maladie, ce qui lui permettra de comprendre ce qu’il sait, ce qu’il fait, comment il le fait, mettant en évidence le bien fondé de ses actions. Il exprimera ses attentes en matière d’accompagnement et d’éducation.

L’étape suivante sera de trouver ensemble comment améliorer les modalités de gestion de la maladie, en déterminant les compétences à acquérir ou à mobiliser et les objectifs pédagogiques qui en découlent. Ces objectifs seront précis et réalistes. Le patient devra comprendre leur utilité dans la vie courante et l’intérêt qu’il a de les mettre en application. Il ne devra pas les ressentir trop complexes et donc hors de portée. Il sera intéressant de donner la priorité aux objectifs pédagogiques ayant un impact immédiat sur la vie quotidienne et la santé du patient. Le but est de soulager, mais aussi de mobiliser l’intérêt et la motivation de l’apprenant.

Aux objectifs pédagogiques s’ajouteront les objectifs plus personnels qui relèvent des besoins spécifiques du patient et de son projet. Ceux-ci sont une source de motivation indispensable à l’adhésion du patient au programme. La définition de l’ensemble de ces objectifs débouchera sur un **contrat d’éducation** individualisé et négocié. L’accord du patient portera non seulement sur les objectifs à atteindre, mais aussi sur le choix des moyens à mettre en œuvre et l’organisation de son programme personnalisé (quel type de consultations, fréquence, les dates et heures de RDV, journées collectives).

Pour cette consultation, notre IDE en éducation thérapeutique s’aidera d’un guide d’entretien facilitant le recueil des informations et comme support structuré du dialogue.

GUIDE D’ENTRETIEN DU DIAGNOSTIC EDUCATIF

***Evaluation des aptitudes physiques, sensorielles, cognitives :***

***Mobilité :***

*Douleur : Elocution:*

*Déglutition : Vision :*

*Elimination: Audition :*

***Mémoire, troubles cognitifs****:*

***Autres :***

***Biomédical (1),  sévérité de la maladie, handicap et problèmes de santé, ce qu’il /elle a :***

Depuis combien de temps avez-vous la maladie de Parkinson ? (histoire de la maladie, hospitalisations, douleurs, handicaps divers)

De quelle façon se manifeste votre maladie, quels sont les symptômes qui vous gênent le plus ?

*Avez vous d’autres problèmes de santé, lesquels ?*

***Socioprofessionnel et familial (2), environnement familial, activités, conditions de vie, ce qu’il/elle fait :***

*Comment vous organisez vous au quotidien ? comment se passent vos journées ?*

*Hygiène de vie, sommeil, siestes, habitudes alimentaires, difficultés diverses:*

*Cadre de vie (adaptation aux besoins)  :*

*Maison individuelle (étages) :*

*Appartement (étages, ascenseur) :*

*Foyer logement :*

*Maison de retraite, médicalisée/non médicalisée :*

*Autre :*

*Mode de vie, environnement familial :*

*En collectivité : Seul :*

*En famille, décrire entourage:*

*Activité du conjoint :*

*Enfants ou personnes à charge :*

*Avez-vous modifié vos activités habituelles ? De quelle façon ?*

*Habitudes de vie :*

*Activités physiques :*

*Loisirs, occupations, vacances :*

*Mode de déplacement, voyage :*

*Vie associative :*

*Profession : Situation sociale :*

*En activité : Arrêt maladie :*

*Poste aménagé : Longue maladie :*

*Demandeur d’emploi : Aides (MDPH):*

*Retraité : ALD :*

*Bénéficiez-vous d’aides sociales spécifiques ? lesquelles, vécu des incapacités, préservation ou réaménagement des activités professionnelles*

***Socioprofessionnel et familial (2), environnement familial, activités, conditions de vie*** *:*

*Comment vous organisez-vous lors des périodes difficiles ?*

*(Gestion des fluctuations de l’état moteur dans la vie quotidienne, préservation de l’autonomie)*

*Qu’avez vous essayé de mettre en place pour améliorer votre situation, pour vous sentir mieux ?*

*(Modifications des habitudes de vie, aménagement du logement, recours à aides techniques, stratégie d’adaptation)*

*Avez-vous fait appel à des aides extérieures ?*

*(Prise de décision, auto-estimation de ses difficultés, niveau d’information sur les aides disponibles, quelles aides)*

*A qui vous adressez-vous pour vos problèmes de santé ? A quel moment ?*

*(Prise de décision, estimation des difficultés, choix de l’intervenant, généraliste, neurologue, autre)*

***Cognitif (3), connaissances, représentations et croyances en matière de santé, de traitement, ce qu’il/elle sait :***

*Pouvez-vous me dire ce que vous savez de cette maladie ?*

*Selon vous, à quoi est due votre maladie ?*

*Connaissez-vous d’autres personnes qui ont cette maladie ?*

*Quels effets ont les traitements que vous prenez ? (description, catégories, perception, croyances)*

*Comment prenez vous votre traitement ? Vous arrive t-il d’en oublier ou d’en prendre plus ? (oublis, addiction, gestion des prises, réveil, pilulier, locus, croyances)*

*Quelle perception avez-vous de votre traitement ? (satisfaisant, contraignant, tolérance...)*

Une synthèse du diagnostic éducatif sera faite et soumise à l’équipe d’éducation lors de réunion mensuelle de l’équipe d’éducation.

| **SYNTHESE DU DIAGNOSTIC EDUCATIF**  **Date :**  **Nom: prénom :**  **Médecin référent :** |
| --- |
| Contexte biomédical, sévérité de la maladie, handicap et problèmes de santé, ce qu’il /elle a : |
| Contexte socioprofessionnel, environnement familial, activités, conditions de vie, ce qu’il/elle fait |
| Connaissances, représentations et croyances en matière de santé, de traitement, ce qu’il/elle sait : |
| Contexte psychoaffectif, ressentis, attitudes, acceptation de la maladie, estime de soi et locus, qui il/elle est : |
| Attentes, besoins du patient, projet personnel, quels sont ses projets : |
| Objectifs, compétences à acquérir, ou à développer : |
| Difficultés, facteurs limitants :  Atouts, motivation : |

### **Etape 2-La Consultation Individuelle d’Education**

Chaque consultation est construite autour d’un objectif pédagogique en lien direct avec la ou les compétences à acquérir.

La durée sera variable, entre 30 et 45 mn. Elle dépendra de la complexité de l’objectif de la séance et des possibilités parfois fluctuantes du patient

**Déroulement de la séance** :

- Au début : présentation des objectifs et du déroulement de la séance, validation par le patient ;
- Tenir compte de l’état moteur ou thymique du patient, de ses capacités à se concentrer ;
- Utilisation des connaissances et de l’expérience du participant pour donner du sens à l’apprentissage ;
- Utilisation de techniques de communication centrées sur la personne telles que l’écoute active, l’entretien motivationnel, pour initier un changement chez le patient et soutenir sa motivation ;
- Alternance des outils et des méthodes à choisir selon le thème de la séance (maximum trois) : affiches, classeur-imagier, vidéo, cédéroms, témoignages documentaires, brochures, simulations de gestes et de techniques ;
- Evaluation de la capacité à mettre en pratique les acquis dans la vie quotidienne grâce à des simulations à partir de l’analyse d’une situation (études de cas) ;
- Synthèse de fin de séance faite avec la participation de l’apprenant, elle facilite l’appropriation du contenu de la séance par celui-ci.

**3-Séance d’ETP par téléphone :**

Elle peut être prévue dans le programme d’éducation entre deux séances individuelles, mais peut aussi être organisée à la demande du patient ou de l’infirmière, ponctuellement, lors d’une consultation. Elle portera sur la mise en pratique d’une nouvelle compétence dont l’acquisition est jugée difficile et nécessite un suivi plus rapproché. Elle permettra d’analyser la situation avec le patient, de trouver des solutions aux problèmes rencontrés et de relancer la motivation.

Ce type de séance fera l’objet d’un rendez-vous téléphonique et ne sera pas à la charge du patient. Elle sera suivie d’une consultation individuelle, soit pour renforcer l’apprentissage selon le niveau de difficultés rencontré, soit pour poursuivre la prise en charge éducative. Elle sera complétée d’un compte rendu dans le dossier éducatif.

**4-Sessions collectives, les Journées à Thème :**

Les sessions collectives seront proposées aux patients qui bénéficient du programme d’éducation. Elles feront l’objet d’un travail spécifique en fonction du thème retenu, les thèmes seront sélectionnés en tenant compte des objectifs pédagogiques des participants.

Ces sessions auront lieu environ tous les deux mois, au cours d’une hospitalisation de jour, de 9h00 à 16h3O. Elles accueilleront 6 à 8 patients en moyenne, ainsi que les proches qui souhaitent y assister. L’animation sera assurée par l’IDE qui accompagnera le groupe toute la journée et par un médecin de l’équipe d’éducation.

Elles seront structurées de la façon suivante :

**Séance de présentation** (durée : 30min) : Accueil, présentation du programme (durée, sujets, horaires, pauses, repas, localisation des toilettes), des intervenants. Tour de table de présentation : prise de contact rapide entre les patients et avec les intervenants présents.

**Séances éducatives** de 40 à 45 min, séparées par des pauses d’au moins 10 min donnant aux patients la possibilité de se mobiliser, de prendre leurs médicaments. Environ trois par journée.

En début d’après midi, une mise en situation avec étude de cas concrets sera proposée en **séance de synthèse** et d’évaluation. Elle permet de faire le point sur la compréhension des points clés transmis pendant la journée et prépare le transfert des acquis dans la vie quotidienne. Un questionnaire de satisfaction sera distribué pendant la dernière pause avant la séance Atelier d’Explicitation qui clôturera la journée.

**L’Atelier d’Explicitation** permettra d’exprimer le ressenti de la journée, de poser les questions que les participants n’ont pas osé poser.

Une consultation individuelle sera organisée environ un mois après la formation, pour évaluer l’acquisition des connaissances et des compétences enseignées, vérifier la mise en pratique, éventuellement revoir les notions mal comprises.

**4-La Consultation Individuelle d’Evaluation** a pour but de mettre en évidence et en valeur les transformations obtenues en termes de compétences et d’amélioration de la qualité de vie.

Elle est proposée à 6 mois de suivi, et plus tôt si nécessaire que ce soit à la demande du patient ou du professionnel de santé. Cette consultation débouche sur l’actualisation du diagnostic éducatif, des objectifs pédagogiques et éventuellement sur la modification du contenu du programme proposé au début de la prise en charge.

Cette évaluation porte sur les éléments suivants :

- Evaluation formative des compétences acquises, à maintenir, à soutenir, à compléter ;
- Evaluation de la qualité de vie, de l’estime de soi, de l’acceptation de la maladie ;
- Evaluation du diagnostic éducatif : les objectifs pédagogiques ont-ils répondu aux attentes du patient, sont-ils toujours adaptés à ses besoins, à l’évolution de la maladie, aux changements de traitement, aux modifications survenues dans sa vie professionnelle, familiale ou affective, dans ses projets de vie.
- Evaluation de la satisfaction du patient : elle permettra de recueillir son ressenti sur la prise en charge éducative qu’il reçoit et en apporter les corrections nécessaires, mais aussi de connaître le point de vue du patient sur le programme d’ETP et sur sa pertinence, les difficultés qu’il a rencontrées, d’évaluer l’organisation et le déroulement des séances.

Le même type d’évaluation aura lieu à la fin du programme. Elle fera le point sur l’ensemble des apprentissages et le niveau d’autonomie de l’apprenant. Elle se clôturera par la négociation avec le patient d’un contrat de sortie du programme et la définition d’objectifs individuels à poursuivre à domicile.

- 1. Critères de jugement

### Critère principal

**Auto-questionnaire de qualité de vie spécifique de la maladie de Parkinson**

La qualité de vie sera évaluée grâce à une échelle spécifique (PDQ-39)

Nous utiliserons l'auto-questionnaire de mesure spécifique PDQ-39 (Parkinson's Disease Quality of Life) (De Boer et al, 1996). Les indicateurs spécifiques s’avèrent aptes à détecter des variations de la qualité de vie observables dans une pathologie donnée, et ne permettent pas en principe les comparaisons entre programmes de soins qui s’adressent à des pathologies différentes (Carrere, 1997). Le PDQ-39 consiste en 39 questions en rapport avec la santé, auxquelles il faut répondre rétrospectivement sur une période de 1 mois en utilisant 5 niveaux de fréquence (toujours, souvent, parfois, rarement, jamais). Cet instrument de mesure de qualité de vie spécifique évalue 8 dimensions de la santé des parkinsoniens : la mobilité, les activités de la vie quotidienne, le bien-être affectif, la gêne psychologique, le soutien social, les troubles cognitifs, la communication et l’inconfort physique.

- 1. Critère(s) secondaire(s)

### Auto-questionnaire de qualité de vie généraliste

Nous utiliserons l'auto-questionnaire de mesure généraliste SF-36 (Medical Outcomes Study 36-item Short Form) (Ware et al, 1992). Le SF-36 comprend 36 items répartis en 8 dimensions : le fonctionnement physique, les limitations dus aux problèmes physiques, l’émotion, le fonctionnement social, la santé mentale, l’énergie, la douleur et la perception de la santé. Cet autoquestionnaire a déjà été utilisé dans la maladie de Parkinson (Riazi et al, 2003, Brefel-Courbon et al, 2003). Il permet d’évaluer la qualité de vie des patients Parkinsoniens et permet la comparaison de cet état par rapport à d’autres populations (population générale ou patients atteints de maladie chronique).

### Echelle motrice : Unified Parkinson's Disease Rating Scale (Fahn et al, 1987)

Les patients parkinsoniens feront l'objet d'une évaluation de leur état moteur selon la grille de l'Unified Parkinson's Disease Rating Scale (UPDRS). L'échelle comporte les 6 sections suivantes : " état mental, comportemental et thymique" (section I), "activités de la vie quotidiennes en périodes on et off" sur la base de l'interview du patient (section II), "examen moteur en période on ou off" selon l'état du patient lors de la visite (section III), "complications du traitement" (section IV), "score modifié de Hoehn et Yahr" (section V), "échelle modifiée de Schwab et England concernant les activités de la vie quotidienne" (section VI).

### **L’évaluation de la dépression et de l’anxiété grâce à l’échelle HAD (Hospital Anxiety and Depression scale).**

L’état psychologique des patients sera évalué grâce à un auto-questionnaire comprenant 14 items (Lepine et al, 1985). Cet auto questionnaire permet d’évaluer les dimensions anxieuses et dépressives ressentis au cours des semaines passées et s’avère suffisamment sensible pour suivre l’évolution de ces troubles au décours d’une intervention (médicaments ou autres).

### Evaluation adaptation sociale-

L'échelle SAS-SR (Social Adjustment Scale Self-Report) est une méthode simple d'évaluation, par autoquestionnaire, de l'adaptation sociale du patient, notamment lors des états dépressifs. Par rapport aux échelles qui reposent sur une interview du patient, l'échelle SAS-SR est plus sensible aux modifications de l'état clinique. L'échelle SAS-RS peut contribuer au diagnostic des états dépressifs mêmes minimes, au suivi clinique après traitement et faire partie des critères d'évaluation des études longitudinales (Achard et al, 1995).

### Coûts

Les coûts seront recueillis de manière prospective grâce à un carnet de consommation de soins remis au patient qu’il devra remplir et qui sera vérifié au cours de chaque visite par l’investigateur (cf carnet de consommations de soins ci-joint).

Le point de vue de la Caisse Primaire d'Assurance Maladie (CPAM) sera adopté pour mesurer les coûts directs correspondant à la valeur des ressources consommées liées à la prise en charge de la pathologie. Nous distinguerons :

**-** les *coûts directs médicaux* recouvrant différents aspects telles la consommation médicamenteuse et l'utilisation de ressources médicales (hospitalisations, consultations et visites de médecins, examens de laboratoires et explorations),

**-** les *coûts directs non médicaux* ayant trait au transport du patient dans le cadre de sa prise en charge médicale

Les coûts seront exprimés en euros. La situation individuelle des patients assurés sociaux au regard des règles d’exonération du ticket modérateur sera prise en considération. Le coût du séjour hospitalier en court séjour sera mesuré à l’aide du tarif en vigueur du Groupe Homogène de Séjour selon les règles du PMSI (Programme de Médicalisation des Systèmes d’Information). Le coût du séjour hospitalier en moyen ou long séjour sera mesuré à l’aide des prix de journée appliqués au nombre de journées d’hospitalisation. Les soins de ville (médicaments, consultations et visites de médecins, examens de laboratoires et explorations) seront valorisés sur la base des montants remboursés par la Sécurité Sociale. Les transports seront valorisés sur la base des montants remboursés par la Sécurité Sociale.

En conclusion, afin de connaître l’impact du programme d’éducation thérapeutique dans la maladie de Parkinson d’un point de vue médico-économique, les dépenses de santé vont être comparées entre les deux groupes de patients au cours de l’année de la réalisation du programme d’ éducation thérapeutique.

Dans ce but, les numéros de sécurité sociale des patients vont être recensés. Les numéros de sécurité sociale des patients permettront d’ accéder aux dépenses liées aux soins de ville (consultations médicales, kinésithérapeute, orthophonie, etc) enregistrées au niveau de la Caisse Primaire d’Assurance Maladie (CPAM) durant la durée du protocole et aux dépenses liées aux hospitalisations.

Grâce à une collaboration avec le Dr Molinier, chef de service du Département d’Information Médicale (DIM) de l’Hôtel Dieu de Toulouse, les données concernant les hospitalisations seront recueillies. Par ailleurs, le Dr Bourrel, de la CPAM, nous aidera à récupérer les données relatives aux soins de ville des patients relevant du Régime Général des Travailleurs Salariés.

Toutes ces informations seront collectées de manière anonyme et les numéros de sécurité sociale ne seront pas utilisés à d’autres fins.

L’approche médico-économique de ce programme est cruciale pour obtenir des arguments économiques nécessaire à sa pérennisation.

### Autres paramètres cliniques

Nous recueillerons aussi les données suivantes :

- les caractéristiques des patients (date de naissance, sexe, niveau scolaire, situation professionnelle actuelle, situation de vie, emploi actuel ou dernier emploi occupé),

- l’histoire de la maladie de Parkinson (dates des premiers symptômes et du diagnostic),

- les co-morbidités associées (date de début et sévérité),

- la consommation médicamenteuse (type de traitement en cours, dose journalière, date de début).

- 1. Population étudiée

### Critères d’inclusion

Seuls les patients répondant aux critères d’inclusion suivant seront retenus :

- Malades des 2 sexes atteints d’une maladie de Parkinson idiopathique répondant aux critères de définition de la UKPDS Brain Bank (United Kingdom Parkinson’s Disease Society Brain Bank Clinical Diagnosis Criteria, Gibb and Lees, 1988) c'est-à-dire présentant une bradykinésie avec au moins l’un des symptômes suivant : rigidité musculaire, tremblement de repos de 4-6 Hz, instabilité posturale

- Malade ne présentant pas de trouble cognitif susceptible de compromettre la qualité de compréhension et de participation du malade au protocole et aux critères de jugement (carnets de bord inclus)

- patient capable de remplir un auto questionnaire

- patient bénéficiant d’un traitement antiparkinsonien, y compris une stimulation cérébrale profonde (depuis plus de 3 mois)

- patient au stade Hoehn et Yahr ≤ 4

- patient ayant donné son consentement libre et éclairé et signé le consentement

- patient affilié à un régime de sécurité sociale.

### Critères de non inclusion

Le patient ne sera pas inclus pour les raisons suivantes :

- Patient présentant un syndrome parkinsonien non idiopathique atypique (de type de type Paralysie SupraNucléaire (PSP), Atrophie Multi systématisée (MSA), Dégénérescence Cortico Basale (DCB), etc…)
- Patients présentant un syndrome parkinsonien induit par un médicament
- Patient déjà inclus dans un essai clinique au moment de l’étude
- Patient au stade Hoehn et Yahr égal à 5
- Patient atteint d’une maladie psychiatrique sévère ou évolutive, ou d’une psychose dopaminergique considérée à l’heure actuelle comme évolutive
- Patient ayant bénéficié depuis moins de 3 mois d’une stimulation cérébrale profonde ou devant en bénéficier dans l’année
- Patient incapable de comprendre le protocole, de remplir un carnet de bord ou un autre critère de jugement ou de suivre les procédures de l’essai clinique
- Patient ayant une détérioration cognitive susceptible d’interférer avec l’évaluation
- Patient sous tutelle, curatelle ou sauvegarde de justice.
  1. Nombre de sujets nécessaires

Si l’on désire une puissance de 80% pour une différence de 4 points de qualité de vie (PDQ-39), compte tenu d’un écart type de 17 points et d’un niveau de 5% dans un test unilatéral, on doit recruter 111 patients Parkinsoniens compte tenu d’un coefficient de corrélation de 50% entre les réponses dans les deux périodes. En tenant compte des sorties d’essais potentielles, on recrutera 120 patients.

Le calcul du nombre de sujets a été réalisé grâce aux données d’une étude utilisant l’échelle de qualité de vie PDQ-39 chez des patients Parkinsoniens (Brefel-Courbon et al, 2003).

- 1. Modalité de recrutement

Les patients atteints de la maladie de Parkinson seront sélectionnés au sein des services de consultation de Neurologie des 2 hôpitaux toulousains (Purpan et Rangueil) par les neurologues spécialisés dans la prise en charge des patients parkinsoniens (Dr Brefel-Courbon, Dr Fabre, Dr Ory-Magne, Pr Rascol) ou dans l’unité des mouvements anormaux du service de Neurologie de l’hôpital Purpan. Cette étude se voulant pragmatique, nous proposerons cette étude de façon consécutive et non sélective à tous les patients parkinsoniens répondant aux critères d’inclusion et d’exclusion.

Nous attendons un recrutement moyen de l’ordre de 5 à 10 patients par mois. La durée des inclusions devrait être de 24 mois.

- 1. Randomisation

La randomisation s’effectuera après la visite d’inclusion. Elle portera sur l’attribution des patients dans l’un des 2 groupes : groupe I : patients bénéficiant des séances d’éducation thérapeutique et groupe II : patient bénéficiant d’un simple suivi médical conventionnel. Elle s’effectue après vérification des critères d’inclusion. Elle doit conduire à l’inclusion du nombre de patients nécessaires dans les groupes étudiés. Cette randomisation sera effectuée par le Centre d’Investigation Clinique de l’hôpital Purpan.

- 1. Déroulement de l’étude

La participation de chaque patient à l’étude est de 12 mois. La durée totale de la période d’inclusion est prévue sur 2 ans.

- 1. Visite d’inclusion

Elle sera réalisée à l’occasion d’une consultation de suivi habituel en Neurologie et comprendra :

- obtention du consentement libre et éclairé du patient

- vérification des critères d’inclusion et d’exclusion

- cotation motrice du patient (UPDRS en condition On pour les patients parkinsoniens)

- réalisation d’un examen médical général et neurologique

- passation des auto questionnaires

- remise du carnet de bord et l’explication des modalités de son remplissage

- Recueil des caractéristiques du patient, des données de sa pathologie (histoire, comorbidités associées), de sa consommation médicamenteuse.

L’ensemble des déplacements hôpital domicile lors des visites de pré inclusion, d’inclusion, et de suivi sera pris en charge par la CPAM car ces visites seront réalisées dans le cadre des visites de suivi de leur pathologie neurologique chronique.

### Visites de suivi et de fin d’étude

Les patients seront évalués lors d’une consultation de suivi habituel de neurologie à 6 et 12 mois après leur inclusion.

Au cours de ces 2 visites, le patient

- bénéficiera d’un examen neurologique avec cotation motrice
- remplira les auto-questionnaires
- remettra le carnet de bord rempli et recevra le carnet de bord pour la visite suivante (pour la visite à 6 mois).
  1. Analyse des données

Les données seront enregistrées à l’aide du logiciel SAS. Le formulaire de saisie informatique correspondra exactement au formulaire papier utilisé pour le recueil des données.

Avant l'exploitation statistique, les données seront rendues anonymes.

L'analyse statistique sera d'une part descriptive et d'autre part inférentielle :

- Les variables qualitatives seront décrites en présentant les fréquences de chacune des modalités de ces variables. Les variables quantitatives seront analysées de manière à établir la médiane, la moyenne, l'écart type et les valeurs extrêmes.

- l’analyse de la qualité de vie se fera classiquement à l’aide d’un modèle linéaire incluant un effet fixe traitement.

Les comparaisons des coûts médicaux, de la symptomatologie motrice et psychique (critères de jugement quantitatifs secondaires) entre les deux traitements se dérouleront aussi selon le processus décrit ci-dessus.

1. Conséquences attendues des résultats du projet

Si l’impact d’un programme d’ETP s’avérait positif en terme de qualité de vie et si il n’engendrait pas de coûts supplémentaires, ce type de prise en charge sera alors généralisé dans la maladie de Parkinson et fera l’objet de communication lors des congrès nationaux et internationaux.

1. Calendrier prévisionnel

Début des inclusions septembre 2008

Fin des inclusions septembre 2010

Fin du suivi des patients septembre 2011

Analyse des résultats décembre 2011

1. Aspects réglementaires et éthiques

Conformément à la loi n° 2004-806 du 9 août 2004 relative à la politique de santé publique et à ses décrets d’application, le projet rentre dans le cadre d’une évaluation de soins courants et l’étude est soumise à l'avis d'un Comité de Protection des Personnes Sud-ouest et Outre-Mer. La recherche ne sera mise en œuvre qu’après l’avis favorable du CPP.

Toutes les informations recueillies sont confidentielles et ne pourront être divulguées. Les participants s'assureront que l'anonymat de chaque sujet participant à l'étude est garanti. Aucune information permettant l'identification des personnes ne sera communiquée à des tiers autres que ceux, représentant du promoteur et du Ministère de la Santé, réglementairement habilités à détenir cette information (et qui sont tenus au secret professionnel).

Les informations recueillies lors de cette étude feront l'objet d'un traitement informatique. Une demande d'autorisation de traitement automatisé des données en matière de recherche dans le domaine de la santé sera effectuée auprès de la CNIL (Commission Nationale de l'Informatique et des Libertés).

Les sujets seront informés des objectifs de l'étude et de leurs droits de refuser d’y participer. Cette étude entrant dans le cadre de l’évaluation des soins courants, son déroulement et la participation ou non du sujet à celle-ci n’interviendront en rien dans sa prise en charge habituelle. Par ailleurs, conformément à l’article 3 de l’arrêté du 9 mars 2007, les sujets seront informés de l’étude par la lettre d’information et pourront manifester leur opposition à participer à cette étude. Il leur sera demandé de signer un accord de participation.

Après le commencement de l’essai, toute modification substantielle de celui-ci doit être soumise au responsable de l’étude. Ce dernier doit obtenir, préalablement à sa mise en oeuvre, un avis favorable du CPP.

1. Bibliographie

**-** Achard S, Chignon JM, Poirier-Littre MF, Galinowski A, Pringuey D, Van Os J, Lemonnier F. [Social adjustment and depression: value of the SAS-SR (Social Adjustment Scale Self-Report). Encephale. 1995;21:107-16.

- Brefel-Courbon C, Desboeuf K, Thalamas C, Galitzky M, Senard JM, Rascol O, Montastruc JL. Clinical and economic analysis of spa therapy in Parkinson's disease. Mov Disord. 2003 ;18:578-84.

**-** De Boer AGEM, Wijker W, Speelman JD, de Haes JCJM**.** Quality of life in patients with Parkinson’s disease: the development of a questionnaire*. J Neurol Neurosurg Psychiatry* 1996; 61: 70-74.

**-** Carrère MO. La qualité de vie liée à la santé et l’évaluation économique. Journal d’Economie Médicale 1997 ; 15 : 5-8.

**-** Fahn S, Elton RL, and members of the UPDRS Development Committee: Unified Parkinson's disease rating scale. In: Recent developments in Parkinson's disease. (Eds Fahn S, Marsden CD, Calne DB, Goldstein M). Florham Park MacHillan Healthcare Information, 1987: 153-163.

**-** Fitzsimmons B, Bunting LB**.** Parkinson’s disease : quality of life issues. Neuroscience nursing 1993 ; 28 :807-818.

**-** Gibb WK, Lees AJ**.** The relevance of the lewy body to the pathogenesis of the idiopathic Parkinson's disease. *J Neurol NeuroSurg Psychiatry* 1988; 51: 745-752.

- [Grosset KA, Grosset DG.](http://www.ncbi.nlm.nih.gov/pubmed/17634109?ordinalpos=2&itool=EntrezSystem2.PEntrez.Pubmed.Pubmed_ResultsPanel.Pubmed_RVDocSum) Effect of educational intervention on medication timing in Parkinson's disease: a randomized controlled trial. BMC Neurol. 2007 16;7:20.

-Karlsen KH, Larsen JP, Tandberg E, Maeland JG. Influence of clinical and demographic variables on quality of life in patients with Parkinson’s disease. J Neurol Neurosurg Psychiatry 1999; 66 : 431-435.

- Lépine JP, Godchau M, Brun P. Anxiety and depression in patients. Lancet, 1985, ii, 1425-1426.

- Macht M, Gerlich C, Ellgring H, Schradi M, Rusiñol AB, Crespo M, Prats A, Viemerö V, Lankinen A, Bitti PE, Candini L, Spliethoff-Kamminga N, de Vreugd J, Simons G, Pasqualini MS, Thompson SB, Taba P, Krikmann U, Kanarik E. Patient education in Parkinson's disease: Formative evaluation of a standardized programme in seven European countries. Patient Educ Couns. 2007 ;65 :245-52.

- [Mercer BS](http://www.ncbi.nlm.nih.gov/sites/entrez?Db=pubmed&Cmd=Search&Term="Mercer BS"%5BAuthor%5D&itool=EntrezSystem2.PEntrez.Pubmed.Pubmed_ResultsPanel.Pubmed_RVAbstractPlus). A randomized study of the efficacy of the PROPATH Program for patients with Parkinson disease. [Arch Neurol.](javascript:AL_get(this, 'jour', 'Arch Neurol.');) 1996 ;53 :881-4.

- [Montgomery EB Jr](http://www.ncbi.nlm.nih.gov/sites/entrez?Db=pubmed&Cmd=Search&Term="Montgomery EB Jr"%5BAuthor%5D&itool=EntrezSystem2.PEntrez.Pubmed.Pubmed_ResultsPanel.Pubmed_RVAbstractPlus), [Lieberman A](http://www.ncbi.nlm.nih.gov/sites/entrez?Db=pubmed&Cmd=Search&Term="Lieberman A"%5BAuthor%5D&itool=EntrezSystem2.PEntrez.Pubmed.Pubmed_ResultsPanel.Pubmed_RVAbstractPlus), [Singh G](http://www.ncbi.nlm.nih.gov/sites/entrez?Db=pubmed&Cmd=Search&Term="Singh G"%5BAuthor%5D&itool=EntrezSystem2.PEntrez.Pubmed.Pubmed_ResultsPanel.Pubmed_RVAbstractPlus), [Fries JF](http://www.ncbi.nlm.nih.gov/sites/entrez?Db=pubmed&Cmd=Search&Term="Fries JF"%5BAuthor%5D&itool=EntrezSystem2.PEntrez.Pubmed.Pubmed_ResultsPanel.Pubmed_RVAbstractPlus). Patient education and health promotion can be effective in Parkinson's disease: a randomized controlled trial. PROPATH Advisory Board .[Am J Med.](javascript:AL_get(this, 'jour', 'Am J Med.');) 1994 ;97 :429-35

**-** Petit H, Alain H, Vermersch P. La maladie de Parkinson, clinique et thérapeutique. Edittion Masson, Paris, 1994, 7- 10.

- Rajendran PR, Thompson RE, Reich SG. The use of alternative therapies by patients with Parkinson’s disease. Neurology 2001; 57 : 790-794.

- Riazi A, Hobart JC, Lamping DL, Fitzpatrick R, Freeman JA, Jenkinson C, Peto V, Thompson AJ. Using the SF-36 measure to compare the health impact of multiple sclerosis and Parkinson's disease with normal population health profiles. J Neurol Neurosurg Psychiatry. 2003 ;74 :710-4.

- Shimbo T, Goto M, Morimoto T, Hira K, Takemura M, Matsui K, Yoshida A, Fukui T. Association between patient education and health-related quality of life in patients with Parkinson's disease. Qual Life Res. 2004 ;13 :81-9.

- Schrag A, Jahanshahi M, Quinn N. How does Parkinson's disease affect quality of life? A comparison with quality of life in the general population. Mov Disord. 2000 ;15 :1112-8.

- [Simons G, Thompson SB, Smith Pasqualini MC; Members of the EduPark consortium.](http://www.ncbi.nlm.nih.gov/pubmed/16781881?ordinalpos=3&itool=EntrezSystem2.PEntrez.Pubmed.Pubmed_ResultsPanel.Pubmed_RVDocSum) An innovative education programme for people with Parkinson's disease and their carers. Parkinsonism Relat Disord. 2006 ;12 :478-85.

**-** Schipper H, Clinch JJ, Olweny CLM. Quality of Life Studies : definitions and conceptual issues. *Quality of Life and Pharmacoeconomics in Clinical Trials*. Second Edition, edited by B. Spilker. Lippincott-Raven Publischers, Philadelphia ,1996.

- Tison F. Maladie de Parkinson et syndromes parkinsoniens. In : La maladie de Parkinson. Acanthe, Masson, SmithKline Beecham, Paris, 1998 : 41-61.

- Ware J, Sherbourne C. The MOS 36-item Short Health Survey 1 : conceptual frame work and item selection. Med Care 1992; 30 : 473-483.

1. ANNEXES

Annexe 1 : Notice d’information et accord de participation

Annexe 2 : Accord de participation

Annexe 3 : Addendum à la notice d’information de l’étude

- 1. Annexe 1 :

**ETUDE**

**« EVALUATION D’UN PROGRAMME D’EDUCATION THERAPEUTIQUE DANS LA MALADIE DE PARKINSON. «**

**Etude ETPARK.**

Madame, Monsieur,

**L’objectif de ce document est de vous fournir toutes les informations qui vous permettront de comprendre le but de cette étude, ainsi que ses contraintes et l’intérêt qu’elle peut présenter pour vous. N’hésitez pas à demander à votre médecin l’explication des mots ou expressions que vous ne comprendriez pas.**

Vous venez d’être hospitalisé(e) ou de bénéficier d’une consultation au CHU de Toulouse dans le service de Neurologie pour une maladie de Parkinson. Votre médecin vous propose de participer à une étude sur la qualité des soins dont l’objectif est mieux connaître cette maladie, d’étudier la manière dont elle est traitée dans le but d’une meilleure prise en charge.

Cette étude est réalisée conformément à la Loi 2004-806 relative à la politique de santé Publique (Articles L.1121-1 à 1126-7 du Code de la Santé Publique). Elle a reçu un avis favorable du Comité Protection des Personnes (CPP) Sud-Ouest et Ouest Mer II en date du 6 novembre 2008*.*

Cette enquête n’a pas d’influence sur la prescription médicale et la prise en charge de votre maladie au cours de votre hospitalisation ni dans le suivi à plus long terme. Votre médecin traitant est libre de modifier ou non les traitements que vous recevez. La durée de votre participation à cette étude est de 1 an.

Les traitements antiparkinsoniens ne sont que symptomatiques et parfois insuffisants. Un programme d’éducation thérapeutique en complément du traitement médicamenteux antiparkinsonien habituel, pourrait exercer une action bénéfique sur les différentes composantes de la maladie et donc améliorer la qualité de vie du Parkinsonien.

L’objectif de cette étude est de montrer une amélioration de la qualité de vie de patients parkinsoniens bénéficiant d’un programme d’éducation thérapeutique par rapport à des patients parkinsoniens n’en bénéficiant pas.

Nous déterminerons par tirage au sort 2 groupes de patients parkinsoniens : le premier groupe aura d’un programme d’éducation thérapeutique pendant 12 mois en association avec son traitement antiparkinsonien habituel et le second groupe aura son traitement antiparkinsonien habituel sans programme d’éducation thérapeutique.

Le programme d’éducation thérapeutique est destiné à aider les patients à acquérir ou maintenir les compétences dont ils ont besoin pour gérer au mieux leur vie avec une maladie chronique. Ceci a pour but de les aider (ainsi que leur famille) à comprendre leur maladie et leur traitement dans le but de les aider à maintenir et améliorer leur qualité de vie. Ce programme comprendra une consultation initiale puis des consultations de suivi individuel (1 à 3 par trimestre), une séance thématique en groupe (2 à 3 par semestre) puis une consultation d’évaluation au bout de 12 mois.

Au sein de chaque groupe, nous évaluerons la qualité de vie grâce des auto-questionnaires. Ces auto-questionnaires seront remplis au début de l’étude puis à 6 et 12 mois au cours d’une consultation habituelle de suivi en Neurologie. 30 minutes environ sont nécessaires pour remplir les autoquestionnaires.

Nous recueillerons aussi les coûts médicaux grâce à un carnet de consommation de soins spécifiant médicaments, hospitalisations, consultations que nous vous demanderons de remplir régulièrement à la maison.

Si vous acceptez de participer à cette enquête, aucun prélèvement sanguin ne sera effectué en dehors des prélèvements que l’on effectue dans le cadre habituel des soins.

Vous êtes libre d’accepter ou de refuser de participer à cette recherche. Si vous refusez de participer, cela n’aura aucune conséquence sur votre prise en charge habituelle.

Vous êtes libre de vous retirer de cette étude à tout moment sans encourir aucune responsabilité, ni aucun préjudice de ce fait.

Tous les résultats obtenus dans cette recherche resteront confidentiels, en accord avec les règles de la loi du 1^er^ juillet 1994 relative au traitement automatisé des données de santé. Vous avez bien noté que votre droit d’accès et de rectification prévu conformément à la loi Informatique et des Libertés (*loi n° 2004-801 du 6 août 2004 modifiant la loi n° 78-17 du 6 janvier 1978 relative à l’informatique, aux fichiers et aux libertés*) pourra s’exercer à tout moment auprès du Docteur Brefel-Courbon, Service de Neurologie A, CHU Purpan, Toulouse (Tel : 05 61 77 75 16).

Docteur Brefel-Courbon

Date de remise de la présente notice : …. / ………../…………….

- 1. Annexe 2 : ACCORD DE PARTICIPATION

De M, M^me^ ……………………………………..………………… (Nom, prénom du patient)

Adresse :………………………………………………………………….…

Rue :…………………………………………………………………………

Code postal :…………………………………..Ville : …………………………………………

Téléphone : Fixe : ……………………………………………………………………

Le Docteur …………………………….. m’a proposé de participer à une étude intitulée :

« **« EVALUATION D’UN PROGRAMME D’EDUCATION THERAPEUTIQUE DANS LA MALADIE DE PARKINSON.**

**Etude ETPARK ».**

J'ai pris connaissance des informations ci-dessus et les ai parfaitement comprises. Il m’a été laissé le temps et le loisir de poser toutes les questions que je souhaitais au sujet de l’enquête, et une réponse a été fournie à toutes mes questions. La teneur et la signification de ces informations m’ont été expliquées avec précision. Il m’a été précisé que je suis libre d’accepter ou de refuser cette enquête. De même, je suis libre de me retirer de cette étude à tout moment sans encourir aucune responsabilité, ni aucun préjudice de ce fait. Cela ne change en rien mes relations avec mon médecin pour mon traitement et mon suivi médical.

**J’accepte librement de participer à cette enquête dans les conditions précisées dans ce document.**

Mon consentement ne décharge pas les médecins de leurs responsabilités. Je conserve tous mes droits garantis par la Loi. J’accepte que les données enregistrées à l’occasion de cette recherche, puissent faire l’objet d’un traitement informatisé. J’ai bien noté que le droit d’accès et de rectification prévu par la Loi ‘Informatique et Libertés’ (article 40) s’exerce à tout moment. Ces données qui me concernent resteront STRICTEMENT CONFIDENTIELLES. Je pourrai, à tout moment, demander toute information complémentaire au Docteur Brefel-Courbon, service de Neurologie A (Tel 05 61 77 75 16). J'ai bien noté qu’il me sera remis un exemplaire du présent formulaire de consentement signé.

Fait à ……………………………….,

Le ……. / ……. / ……. Le ……. / ……. / …….

Signature du patient  Signature de l’investigateur

- 1. Annexe 3 : Addendum à la Notice d’Information de l’étude

**« Evaluation d’un programme d’éducation thérapeutique dans la maladie de parkinson » - Etude ETPAR**KMadame, Monsieur,

Vous avez accepté(e) de participer à l’étude clinique « Evaluation d’un programme d’éducation thérapeutique dans la maladie de parkinson» pour lequel le Dr Christine Brefel-Courbon est responsable de l’étude. Vous avez déjà reçu un formulaire d’information relatif à cette étude et vous avez signé un consentement pour participer à cette étude.

Cet addendum précise des informations complémentaires sur cette étude. Un des objectifs de l’étude est l’évaluation des coûts médicaux que nous recueillons actuellement à partir du carnet de consommation de soins que vous remplissez régulièrement à la maison. En plus, nous aurions besoin de recenser votre numéro de sécurité sociale qui nous permettra de connaître vos dépenses liées à vos soins de ville (consultations médicales, kinésithérapeute, orthophonie, etc) enregistrées au niveau de la Caisse Primaire d’Assurance Maladie (CPAM) et vos dépenses liées aux hospitalisations. Ceci sera effectué uniquement pour la durée du protocole. Ces informations seront collectées de manière anonyme et votre numéro de sécurité sociale ne sera pas utilisé à d’autres fins.

Toutefois vous n’êtes pas obligé de participer à ce complément d’étude pour poursuivre votre participation à l’étude.

Je soussigné(e), Nom :………………………………Prénom : ……………………........,

après avoir lu cet addendum à la notice d’information et en avoir discuté et obtenu les réponses à toutes mes questions :

- autorise l’accès à mon numéro de sécurité sociale __

- n’autorise pas l’accès à mon numéro de sécurité sociale __

Fait à …………………………..

Le ……. / ……. / ……. Le ……. / ……. / …….

Signature du patient Signature de l’investigateur

*Fait en trois exemplaires : un exemplaire est remis au volontaire, le second exemplaire est conservé par l’investigateur, le 3^ème^ est destiné au promoteur.*
