# Supplementary material for: Clinical Impact and Cost-Effectiveness of an Education Program for PD Patients: A Randomized Controlled Trial
Source: PLoS One. 2016 Sep 29;11(9):e0162646. doi: 10.1371/journal.pone.0162646 (PMC5042480; doi:10.1371/journal.pone.0162646)
Supplement: S2 Protocol — (DOC) [file pone.0162646.s005.doc]

| **TITLE**: | **ASSESSMENT OF A THERAPEUTIC EDUCATION PROGRAM FOR PARKINSON’S DISEASE.**  **ETPARK Study** | |
| --- | --- | --- |
| **ORGANIZATION**: | Name,  Address, Telephone: | CHU TOULOUSE  Hôtel Dieu, 2 rue Viguerie, TSA 80035  31 059 Toulouse Cedex 9 – France  Contact: Llau Marie-Elise  Tel.: +33 (0)5 61 77 87 71 |
| **STUDY PHYSICIAN**: | Name,  Address, Telephone: | Dr. Christine Brefel-Courbon  Neurology Unit, Neuroscience Center,  CHU Toulouse  Tel.: +33 (0)5 61 14 75 16  E-mail: brefel@cict.fr |
| **ASSOCIATE PHYSICIANS**: | Name,  Address: | Dr. Fabienne Ory, Neurology Unit,  Neuroscience Center, CHU Toulouse  Dr. Nelly Fabre, Neurology Unit,  Neuroscience Center, CHU Toulouse  Prof. Olivier Rascol, Neurology Unit,  Neuroscience Center, CHU Toulouse  Prof. Michel Clanet, Neuroscience Center Coordinator, CHU Toulouse  Tel.: +33 (0)5 61 14 75 16 |
| **OTHER PARTICIPANTS:** | Name,  Address: | Prof. Claire Thalamas (logistics and methodological support)  Clinical Investigation Center, CHU Toulouse  Tel.: +33 (0)5 61 77 91 03  Dr. Gérard TAP- biostatistician,  Clinical Investigation Center, CHU Toulouse  Céline Arcari, IDE,  Neuroscience Center, CHU Toulouse  Dr. Laurent Molinier (Medical Information Department at CHU Toulouse)  Dr. Robert Bourrel (CPAM) |
| **TYPE OF STUDY**: | *Open, randomized study* | |
| **DURATION OF THE PROJECT**: | 3 years | |
| **NUMBER OF SUBJECTS**: | 120 patients | |
| **PUBLIC HEALTH LAW** of 9 August 2004 | YES, research aimed at assessing intermediate care. | |
| **Registration No.**: | 2008-A00720-55 | |
| **KEYWORDS**: | Parkinson’s, therapeutic education, quality of life, medico-economic study. | |

1. ABSTRACT

**CONTEXT OF THE RESEARCH PROJECT:**

Antiparkinsonian treatments are only symptomatic and are often insufficient, especially when the disease is progressive. A therapeutic education program (TEP) accompanying the usual medical antiparkinsonian treatment could have a beneficial action on the various components of the disease and thus improve the Parkinson’s patient’s quality of life.

A few publications currently exist that talk about the interest of therapeutic education in Parkinson’s disease, reporting an improvement in certain aspects of quality of life, psychological condition and treatment compliance among Parkinson’s patients. As far as we know, no study of this kind has been carried out in France.

**OBJECTIVES OF THE RESEARCH PROJECT**:

The main objective of the study is to demonstrate the improvement in quality of life among Parkinson’s patients who benefit from a TEP program compared with Parkinson’s patients who do not benefit from such a program. The secondary objectives of the study are to compare the motor condition, psychological condition, social adjustment and medical costs in the two groups of Parkinson’s patients.

**METHODS AND TOOLS:**

The study is an open, randomized, monocentric, comparative prospective study of 2 groups of Parkinson’s patients (the first following a TEP program for 12 months, the second not following this program). We will assess the quality of life of Parkinson’s patients using a specific scale (PDQ-39) and a general scale (SF-36) at 6 and 12 months in 120 Parkinson’s patients (60 in each group). We will also assess the motor condition, psychological condition and social adjustment of the two patient groups. We will gather figures on medical costs using a healthcare consumption notebook indicating the drugs taken, hospitalizations, consultations, etc.

**CONDUCT AND DURATION OF THE PROJECT**:

Within each group, the various parameters will be gathered at the baseline and then at 6 and 12 months during a normal neurology follow-up consultation. The TEP program will include an initial consultation, then individual follow-up consultations (1 to 3 per three-month period), thematic group sessions (2 or 3 per six-month period), and then an assessment consultation at the end of the 12-month period. The inclusion period will last 24 months and the total duration of the study will be 36 months.

**IMPACT OF THE EXPECTED RESULTS:**

The results expected are an improvement in the quality of life among the Parkinson’s patients who follow a TEP program compared with the Parkinson’s patients who do not follow such a program.

We hypothesize that there will be a cost differential in favor of the Parkinson’s patients who follow a TEP program.

1. SCIENTIFIC JUSTIFICATION AND GENERAL DESCRIPTION

Parkinson’s disease, with a prevalence of 150/100,000 inhabitants, is the second leading cause of neurodegenerative disorders, behind Alzheimer’s disease (Petit et al., 1994). It concerns approximately 2/3 to 3/4 of all Parkinsonian syndromes and affects approximately 1.5% of all subject over the age of 60 (Tison, 1998). A chronic, degenerative neurological disease, it is characterized not only by a motor symptomatology (akinesia, stiffness, rest tremor, postural anomalies) but also cognitive and psychological disorders, sleep disturbances, pain and sexual dysfunction (Fitzsimmons et al., 1993). This varied symptomatology can therefore affect certain aspects of everyday life. Several studies have shown that there is an alteration of the quality of life with Parkinson’s disease (Karlsen et al., 1999; Schrag et al., 2000; Riazi et al., 2003). Antiparkinsonian treatments are only symptomatic and often insufficient, especially when the disease is progressive. Nearly half of all Parkinson’s patients use at least one alternative therapy (Rajendran et al., 2001).

A therapeutic education program, alongside the usual antiparkinsonian treatment, could have a beneficial action on the various components of the disease and thus improve the quality of life of Parkinson’s patients.

A few publications currently exist that talk about the interest of therapeutic education in Parkinson’s disease (Simons et al., 2006; Shimbo et al., 2004; Montgomery et al., 1994; Mercer et al., 1996; Macht et al., 2007, Grosser et al., 2007). But many of them do not assess therapeutic education with a sufficient methodology (Simons et al., 2006; Shimbo et al., 2004; Macht et al., 2007). Indeed, these studies do not include a control group and only demonstrate feasibility and the satisfaction felt by the patients and their caregivers. Three randomized comparative studies carried out in the United States and the United Kingdom reported an improvement in certain aspects of quality of life, psychological condition and treatment compliance in Parkinson’s patients (Montgomery et al., 1994; Mercer et al., 1996; Grosser et al., 2007). As far as we know, no study of this kind has been carried out in France.

In France, we can nonetheless take inspiration from the experience acquired by diabetologists concerning the interest of therapeutic education in treating diabetes. In 2002, the ANAES (Agence nationale d'accréditation et d'évaluation en santé – National Healthcare Accreditation and Assessment Agency) validated therapeutic education as a necessary, integral part of care for diabetic patients. Diabetes, like Parkinson’s disease, is a chronic disease in which intervention by the patient is by no means insignificant.

Parkinson’s disease in everyday life requires the people affected (and/or their caregivers):

- to take part personally in the decisions that will influence the effectiveness of their treatment (diet, physical activity, body maintenance and control, self-monitoring of symptoms, personal health practices, adequate specialized medical and paramedical appointments, etc.);
- and to ensure prevention or early screening for possible complications, notably iatrogenic effects.

Since 2005, our experiment at the abnormal movement unit of the CHU Toulouse University Hospital Center has shown that weekly meetings with hospitalized patients suffering from Parkinson’s disease, whether accompanied by their spouse or not, under the responsibility of a nurse and a physician aware of the interest of therapeutic education, enables patients to integrate the principles behind their treatment, the therapeutic outlook for the future, and the prognosis for their disease. This pilot experiment leads to a better acceptance of their disease, better treatment compliance and easier relations with caregivers and physicians.

Lastly, the current economic context makes it necessary to assess the cost of this kind of care. It is possible that an improvement in the quality of life and a better management of care for their chronic disease may lead to a reduction in healthcare consumption.

It is therefore important to undertake a medico-economic study in order to determine the impact of a therapeutic education program in Parkinson’s disease.

**Therapeutic education for patients (TEP)**

According to the WHO (1996), therapeutic education for patients aims at helping patients to acquire or maintain the skills they need to manage their life with a chronic disease as well as possible. This is a permanent, integral part of the patient’s care. It includes organized activities, including psychosocial support designed to keep patients’ aware and informed of their disease, their care, hospital procedures and organization and the behaviors related to healthcare and the disease. The goal is to help them (as well as their families) to understand their disease and their treatment, to work together and shoulder their responsibilities in their own care with a view to helping them to maintain and improve their quality of life.

The purposes of TEP include:

- the patient’s acquisition and maintenance of self-care skills (adapting drug doses to the motor condition, performing technical healthcare gestures, undertaking a program of physical activity, etc.);

- acquisition of psychosocial adjustment skills (knowing how to manage one’s emotions and managing one’s stress toward Parkinson’s disease, etc.).

TEP is therefore designed for all Parkinson’s patients, no matter what the stage of their disease, and for the people around them:

- because it is complementary and inseparable from their treatment and care, relieving their symptoms and preventing complications.

- because it contributes to improving patients’ health condition (biological and clinical) and their quality of life as well as that of those around them.

This TEP program is drawn up with the patient and personalized for his/her needs (personal or therapeutic). It facilitates or organizes interventions by other healthcare professionals such as psychologists, physiotherapists, social workers, dieticians, occupational therapists, etc.

This TEP program is undertaken:

- after announcing the diagnosis, to help understand, reformulate and provide psychological support;
- during the patient’s regular follow-up consultation, to consolidate skills, make updates for better compliance, good management of side effects and optimize quality of life;
- during an in-depth follow-up consultation, in a difficult period, if a patient is having difficulties with learning.

There are four steps:

- the educational diagnosis, focusing on the patient’s needs (what they have, what they do, what they know, what they think, who they are, what projects they have);
- skills to be acquired, objectives for the patient to reach, therapeutic objectives, contract;
- the educational methods, pedagogical tools (program of consultations, participation in group sessions);
- regular assessment of the progress made: what the patients know, what they know how to do, what they understand, what remains to be learnt, have they made progress in their project.

We therefore want to set up a medico-economic study aimed at assessing the impact of a therapeutic education program on patients afflicted with Parkinson’s disease.

1. OBJECTIVES
   1. Main objective

The main objective of the study is to show improvement in the quality of life among Parkinson’s patients following a therapeutic education program compared with Parkinson’s patients who do not follow such a program.

- 1. Secondary objectives

The secondary objectives of the study are to compare the motor condition, psychological condition, social adjustment and medical costs of two groups of Parkinson’s patients.

- 1. Research hypothesis and results expected

The results expected are an improvement in the quality of life of Parkinson’s patients who follow a therapeutic education program compared with Parkinson’s patients who do not follow such a program.

This improvement in quality of life may be correlated with an improvement in the psychological condition and/or motor condition.

We hypothesize that there will be a cost differential in favor of the Parkinson’s patients who follow a therapeutic education program.

1. METHODOLOGY
   1. Experimental plan

The study is an open, randomized, monocentric, comparative prospective study of 2 groups of Parkinson’s patients (the first following a therapeutic education program, the second not following this program).

The Parkinson’s patients will be randomly assigned to group I (patients following the therapeutic education program) and group II (patients not following the therapeutic education program).

In both groups, patients will undergo a clinical assessment before randomization and after 6 and 12 months of inclusion. This assessment calls for completing a self-assessment questionnaire that aims to assess quality of life, anxiety, depression and social consequences for these patients. Furthermore, at each visit, neurologists specialized in Parkinson’s disease will carry out a clinical examination notably including a motor assessment. Lastly, at each visit, the patients will give hand in a notebook in which they systematically note each medical consultation (specialized or not) and each hospitalization in order to undertake a medico-economic assessment.

Patients in both groups will undergo regular, conventional follow-ups by their referring neurologist.


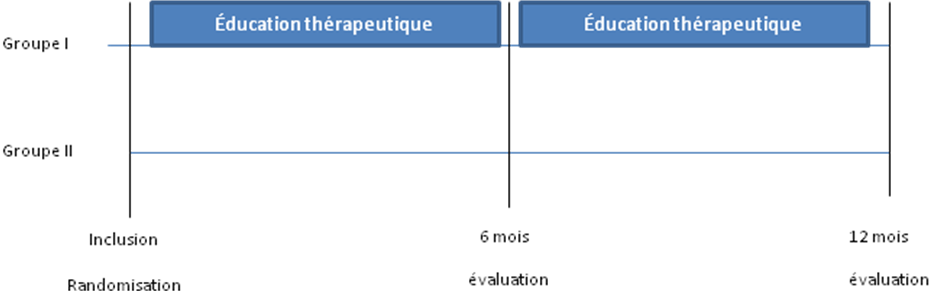


|  | Visit 1: 1^st^ month | Visit 2: 6^th^ month | Visit 3: 12^th^ month |
| --- | --- | --- | --- |
| Informed consent | X |  |  |
| Randomization | X |  |  |
| Self-assessment questionnaires | X | X | X |
| Clinical examination | X | X | X |
| Filling in the notebook | X | X | X |

We have chosen to carry out a pragmatic study in order to respect the patient’s usual living conditions. We are therefore seeking to interfere as little as possible in the usual care outside the TEP visits.

- 1. Therapeutic Education Program (TEP)

This Therapeutic Education Program was developed following the recommendations proposed by France’s Haute Autorité de Santé in its guidebook: *Guide Méthodologique-Structuration d’un programme d’éducation thérapeutique du patient dans le champ des maladies chroniques*, (*Methodological Guide-Structuring of a Patient Therapeutic Education Program in the Field of Chronic Diseases*) Haute Autorité de Santé-INPES, June 2007, and according to the recommendations for caring for Parkinson’s patients in the guidebook *Syndromes Parkinsoniens dégénératifs ou secondaires non réversibles (Degenerative or secondary non-reversible Parkinsonian Syndromes)* published by the Haute Autorité de Santé. On this basis, our team, made up of a registered nurse with training in therapeutic education (Céline Arcari) and neurologists specialized in IPD (Drs. Christine Brefel Courbon and Fabienne Ory Magne), drew up a therapeutic education program applied to patients suffering from IPD.

This program is carried out in 3 steps, which are:

### Step 1: The Initial Education Consultation

This precedes any educational intervention. Its content will be recorded in the education file and will remain confidential. It lasts 45 minutes, to be adapted depending on the patient’s possibilities. The Initial Consultation is carried out by Céline Arcari and comprises two steps:

- Establishing the educational diagnosis;
- Establishing the education contract and the patient’s program.

The **educational diagnosis** is the starting point of the education process; it is the first meeting between the patient and the healthcare professional. It enables the healthcare professional to gather precise information by gaining an understanding of the various dimensions of the patient’s life (family data, socioprofessional data, understanding of the disease, representations and beliefs in terms of healthcare, impressions and acceptance of the disease, the patient’s projects).

With this questioning, the healthcare professional gains a better understanding of patients, their personal and social resources, their reactions in confronting the disease, their motivations and blocks to the educational process. Likewise, it will help them to think about their acts and their perception of the situation by helping them to realize what they know and what they do not know.

For patients, the **educational diagnosis** is the start of the education process. It enables them to talk about themselves, their beliefs and their convictions. Patients can express their needs, their fears and their emotions. Through talking about their lives, they describe their experience with the disease, which enables them to understand what they know, what they do and how they do it, highlighting the merits of their actions. They can express their expectations for support and education.

The next step will be to work together to find ways to improve how they manage the disease, determining the skills they need to acquire or mobilize and the resulting educational goals. These goals will be precise and realistic. Patients have to understand their usefulness in everyday life and the interest they have in applying them. They should not feel that they are too complicated and therefore unachievable. It will be interesting to place priority on educational goals that have an immediate impact on the patient’s everyday life and health. The aim is to provide relief, but also to stimulate the learner’s interest and motivation.

Alongside the educational goals, there are more personal goals dealing with the patient’s specific needs and their project. These are an indispensable source of motivation for patients to comply with the program. Defining all these goals will lead to an individualized, negotiated **education** **contract**. The patient’s agreement will not only cover the goals to be met, but also the choice of the means to be used and the organization of the personalized program (what type of consultations, frequency, appointment dates and times, group days).

For this consultation, our therapeutic education nurse will be assisted by an interview guide to be used for gathering information and as a structured support for dialogue.

EDUCATIONAL DIAGNOSIS INTERVIEW GUIDE

***Assessment of physical, sensory and cognitive skills:***

***Mobility:***

*Pain: Speaking:*

*Swallowing: Vision:*

*Elimination: Hearing:*

***Memory, cognitive disorders****:*

***Other:***

***Biomedical aspects (1), severity of the disease, disability and health problems, what he/she has:***

How long have you had Parkinson’s disease? (disease case history, hospitalizations, pain, miscellaneous disabilities).

How does the disease manifest itself, which symptoms bother you the most?

*Do you have other health problems? If so, what ones?*

***Socioprofessional and family aspects (2), family environment, activities, living conditions, what he/she does:***

*How do you organize your everyday life? What are your days like?*

*Personal health practices, sleep, naps, eating habits, miscellaneous difficulties:*

*Living environment (adjustment to needs):*

*Individual home (floors):*

*Apartment (floors, elevator):*

*Assisted living facility:*

*Retirement home, with medical services/non-medical:*

*Other:*

*Way of life, family environment:*

*Collective: Alone:*

*With family, describe family members:*

*Spouse’s activity:*

*Children or dependents:*

*Have you changed your usual activities? In what way?*

*Everyday habits:*

*Physical activities:*

*Leisure, pastimes, vacation:*

*Travel methods, trips:*

*Associations:*

*Profession: Social status:*

*Working: Medical leave:*

*Adapted workstation: Extended medical leave:*

*Job-seeker: Assistance (MDPH – public assisted living facility):*

*Retired: Long-term disorder:*

*Do you receive specific social assistance? What kind? How do you live with disabilities, preservation or adaptation of professional activities?*

***Socioprofessional and family aspects (2), family environment, activities, living conditions:***

*How do you organize your life during difficult periods?*

*(managing fluctuations in motor conditions in everyday life, preserving autonomy)*

*What have you tried to set up to improve your situation, so that you feel better?*

*(changing one’s living habits, adapting your home, use of technical aids, adjustment strategy)*

*Have you called upon outside help?*

*(decision-making, self-assessment of one’s difficulties, amount of information on the aid available, what aid)*

*Who do you go to for your health problems? When?*

*(decision-making, assessment of one’s difficulties, choice of the practitioner, general practitioner, neurologist, other)*

***Cognitive aspects (3), knowledge, representations and beliefs concerning health and treatment, what he/she knows:***

*Can you tell me what you know about this disease?*

*What do you think your disease is caused by?*

*Do you know other people who have this disease?*

*What effects do the treatments you take have? (description, categories, perception, beliefs)*

*How do you take your treatment? Do you sometimes forget to take it or do you take more? (forgetting, addiction, managing doses, getting out of bed, pill organizer, locus, beliefs)*

*How do you feel about your treatment? (satisfactory, constraint, tolerance, etc.)*

A summary of the educational diagnosis is drawn up and submitted to the education team at the monthly education team meeting.

| **SUMMARY OF THE EDUCATIONAL DIAGNOSIS**  **Date:**  **Last name: First name:**  **Referring physician:** |
| --- |
| Biomedical context, seriousness of the disease, disability and health problems, what he/she has: |
| Socioprofessional context, family environment, activities, living conditions, what he/she does |
| Knowledge, representations and beliefs in the area of health and treatment, what he/she knows: |
| Psychoemotional context, impressions, attitudes, acceptance of the disease, self-esteem and locus, what he/she is: |
| Expectations, patient needs, personal project, what projects do they have: |
| Objectives, skills to be acquired or developed: |
| Difficulties, limiting factors:  Assets, motivation: |

### **Step 2 – The Individual Education Consultation**

Each consultation is organized around an educational goal in direct relation with the skills to be acquired.

Its duration may vary between 30 and 45 minutes. This will depend on the complexity of the goal for the session and the patient’s possibilities, which may fluctuate.

**Conduct of the session**:

- Start: presentation of the goals and the conduct of the session, validation by the patient;
- Take the patient’s motor or thymic condition into account, with his/her ability to concentrate;
- Make use of the participant’s knowledge and experience to give meaning to the learning process;
- Use communication techniques that focus on the person, such as active listening and motivational counselling, to initiate a change in the patient and support his/her motivation;
- Alternate between tools and methods to be chosen in relation to the topic of the session (three at most): posters, folder/picture-word books, videos, CD-ROMs, documentary testimonials, brochures, simulations of gestures and techniques;
- Assessment of the patient’s ability to apply the knowledge acquired in everyday life thanks to simulations based on the analysis of a situation (case studies);
- End-of-session summary with the learner’s participation, this helps the learner to make the content of the session his/her own.

**3- Telephone TEP sessions:**

These can be used during the education program between two individual sessions, but can also be organized upon request from the patient or the nurse during a consultation for a one-off session. It will deal with the implementation of a new skill whose acquisition is felt to be difficult and which requires closer monitoring. It will be used to analyze the situation with the patient, to find solutions to the problems that arise and to boost motivation.

This type of session will give rise to a telephone meeting and will not be at the patient’s expense. It will be followed by an individual consultation, either to reinforce the learning process depending on the level of difficulty encountered, or to pursue the educational coverage. It will be rounded out by a report in the education file.

**4-Group sessions, Theme Days:**

Group sessions will be offered to patients who follow an education program. They will give rise to specific work depending on the theme of the day, which will be selected in relation to the participants’ educational goals.

These sessions will be held approximately every two months, during an outpatient visit, from 9:00 am to 4:30 pm. They will include 6 to 8 patients on average, as well as family members or friends who would like to attend. They are hosted by the registered nurse who accompanies the group for the whole day and a physician from the education team.

They will be structured as follows:

**Presentation session** (duration: 30 min.): Welcome, presentation of the program (duration, subjects, times, breaks, meals, location of the rest rooms) and the coordinators. Introduction round: a quick initial contact between the patients and the coordinators who are present.

**Educational sessions** lasting 40 to 45 min., separated by breaks lasting at least 10 min. to give the patients an opportunity to move around and to take their medications. There will be approximately three breaks during the day.

At the start of the afternoon, a simulation with a concrete case study will be proposed in a **wrap-up and assessment session**. This is an opportunity to take stock of how well the key points covered during the day have been understood and to prepare the transfer of the knowledge acquired into everyday life. A satisfaction questionnaire will be distributed during the last break before the Clarification Workshop session that closes out the day.

**The Clarification Workshop** is an opportunity to express one’s feelings about the day and to ask questions that the participants had not dared to ask earlier.

An individual consultation will be organized approximately once a month after the training to assess the acquisition of the knowledge and skills taught, check that they are applied and, if necessary, review those notions that were not fully understood.

**4-The Individual Assessment Consultation** is designed to highlight and promote the transformations obtained in terms of skills and improvement in quality of life.

It is proposed after 6 months of follow-up and earlier if necessary, whether at the patient’s or healthcare professional’s request. This consultation gives rise to an update of the educational diagnosis, the educational goals and, if necessary, a change in the content of the program proposed at the start of care.

This assessment covers the following elements:

- Formative assessment of the skills acquired, to be maintained, to support and to complete;
- Assessment of quality of life, self-esteem, acceptance of the disease;
- Assessment of the educational diagnosis: did the educational goals meet the patient’s expectations, are they still suited to his/her needs, to the progression of the disease, to changes in treatment, to changes in his/her professional, family or emotional life, to his/her life projects.
- Assessment of patient satisfaction: this is used to gather personal feelings about the educational care received and to make any corrections needed, but also to gather the patient’s point of view on the TEP program and on its relevance, the difficulties encountered, and to assess the organization and conduct of the sessions.

The same kind of assessment will be undertaken at the end of the program. It will take stock of all the learning dispensed and the learner’s level of autonomy. It will close with negotiations with the patient on an end-of-program contract and the definition of individual goals to be pursued at home.

- 1. Assessment criteria

### Main criterion

**Self-assessment questionnaire on quality of life specific to Parkinson’s disease**

Quality of life will be assessed using a specific scale (PDQ-39).

We will use the specific self-assessment measurement questionnaire, PDQ-39 (Parkinson's Disease Quality of Life) (De Boer et al., 1996). Its specific indicators are suited to detecting observable variations in the quality of life for a given disease, and in principle cannot be used to make comparisons between care programs that address different diseases (Carrere, 1997). The PDQ-39 is made up of 39 health-related questions that are to be answered retrospectively over a period of 1 month using 5 levels of frequency (always, often, sometimes, rarely, never). This specific quality of life measurement instrument assesses 8 dimensions of Parkinson’s patients’ health: mobility, everyday activities, emotional well-being, psychological stress, social support, cognitive disorders, communication and physical discomfort.

- 1. Secondary criteria

### General quality of life self-assessment questionnaire

We will use the general measurement self-assessment questionnaire, SF-36 (Medical Outcomes Study 36-item Short Form) (Ware et al., 1992). The SF-36 is made up of 36 items divided into 8 dimensions: physical functioning, limitations due to physical problems, emotions, social functioning, mental health, energy, pain and health perceptions. This self-assessment questionnaire has already been used for Parkinson’s disease (Riazi et al., 2003, Brefel-Courbon et al., 2003). It provides an assessment of quality of life among Parkinson’s patients and makes it possible to compare this condition with other populations (general population or patients suffering from a chronic disease).

### Motor scale: Unified Parkinson's Disease Rating Scale (Fahn et al., 1987)

The Parkinson’s patients will undergo an assessment of their motor condition using the Unified Parkinson's Disease Rating Scale (UPDRS) grid. The scale is made up of the following 6 sections: " mental, behavioral and thymic condition " (section I), "activities in everyday life during on and off periods" based on the patient interview (section II), "motor examination during on or off periods" according to the patient’s condition at the time of the visit (section III), "treatment complications" (section IV), "modified Hoehn and Yahr score" (section V), "modified Schwab and England scale concerning activities in everyday life" (section VI).

### **Assessment of depression and anxiety with the HAD (Hospital Anxiety and Depression) scale.**

The patients’ psychological condition will be assessed using a self-assessment questionnaire made up of 14 items (Lepine et al., 1985). This self-assessment questionnaire is used to assess the anxiety and depression aspects felt during the last few weeks and is sufficiently sensitive to monitor the evolution of these disorders during an intervention (medication or other).

### Social adjustment assessment

The SAS-SR (Social Adjustment Scale Self-Report) scale is a simple assessment method using a self-assessment questionnaire applied to the patient’s social adjustment, notably during depressive episodes. Compared with scales based on a patient interview, the SAS-SR scale is more sensitive to changes in the clinical condition. The SAS-RS scale can contribute to the diagnosis of depressive conditions, even slight, and clinical follow-up after treatment; it can be included in the assessment criteria for longitudinal studies (Achard et al., 1995).

### Costs

Cost figures will be gathered prospectively using a healthcare consumption notebook given to the patient who will fill it in. It will be checked by the investigator at each visit (cf. healthcare consumption notebook, attached).

The point of view issued by the CPAM (Caisse Primaire d'Assurance Maladie – France’s Primary Health Insurance Fund) will be adopted to measure the direct costs corresponding to the value of the resources consumed in relation to care for the disease. We will make a distinction between:

**-** *direct medical costs* covering various aspects such as medication consumption and the use of medical resources (hospitalizations, doctor visits and consultations, lab tests and explorations),

**-** *directs non-medical costs* related to the patient’s transportation in the context of medical care.

Costs will be expressed in euros. The individual situation of patients covered by State health insurance concerning the rules for deterrent fee exemptions will be taken into account. The cost of short-term hospital stays will be measured using the applicable rates of the Groupe Homogène de Séjour (Homogenous Hospital Stay Group) following the PMSI (Programme de Médicalisation des Systèmes d’Information - Medicalized Information System Program) rules. The cost of a medium or long-term hospital stay will be measured using the daily prices applied to the number of days of hospitalization. Private practice care (medication, doctor’s visits and consultations, lab tests and explorations) will be valued on the basis of the amounts reimbursed by the French Social Security system. Transportation will be valued on the basis of the amounts reimbursed by the French Social Security system.

In conclusion, to understand the impact of the therapeutic education program in Parkinson’s disease from a medico-economic point of view, healthcare expenses will be compared for the two groups of patients during the year of the therapeutic education program.

For this, the patients’ social security numbers will be gathered. The patients’ social security numbers will be used to gain direct access to the expenses connected to their private practice care (medical consultations, physiotherapists, speech therapy, etc.) recorded at the CPAM (Caisse Primaire d’Assurance Maladie – France’s Primary Health Insurance Fund) during the protocol period and expenses connected to hospitalizations.

Data on hospitalizations will be gathered thanks to collaboration with Dr. Molinier, head of the DIM (Département d’Information Médicale – Medical Information Department) at the Hôtel Dieu in Toulouse. Moreover, Dr. Bourrel of the CPAM will help us to retrieve data relative to private practice care for patients covered by the Régime Général des Travailleurs Salariés (General Scheme for Salaried Employees).

All of this information will be gathered anonymously and the social security numbers will not be used for any other purpose.

This program’s medico-economic approach is crucial to obtaining the economic arguments needed for its continuation.

### Other clinical parameters

We will also gather the following data:

- characteristics of the patients (date of birth, sex, educational level, current job situation, living situation, current job or last job held);

- Parkinson’s disease case history (dates of the first symptoms and diagnosis);

- related comorbidities (start date and severity);

- consumption of medication (type of current treatment, daily dose, start date).

- 1. Study population

### Inclusion criteria

Only patients who met the following inclusion criteria will be accepted:

- Patients of both sexes with idiopathic Parkinson’s disease meeting the criteria in the definition of the UKPDS Brain Bank (United Kingdom Parkinson’s Disease Society Brain Bank Clinical Diagnosis Criteria, Gibb and Lees, 1988), i.e. presenting bradykinesia with at least one of the following symptoms: muscle stiffness, rest tremor at 4-6 Hz or postural instability;

- Patient with no cognitive disorders that could compromise the quality of understanding and participation by the patient in the protocol and the assessment criteria (notebooks included);

- Patient capable of filling in a self-assessment questionnaire;

- Patient benefiting from an antiparkinsonian treatment, including deep brain stimulation (for more than 3 months);

- Patient at a Hoehn and Yahr stage of ≤ 4;

- Patient having given free, informed consent and signed the consent form;

- Patient affiliated with a social security health insurance scheme.

### Exclusion criteria

The patient will not be included for the following reasons:

- Patient with an atypical non-idiopathic Parkinsonian syndrome (such as Progressive Supranuclear Palsy (PSP), Multiple System Atrophy (MSA), Corticobasal Degeneration (CBD), etc.);
- Patients with a Parkinsonian syndrome induced by a drug;
- Patient already included in a clinical trial at the time of the study;
- Patient at a Hoehn and Yahr stage of 5;
- Patient with a severe or progressive psychiatric illness or dopaminergic psychosis currently considered as being progressive;
- Patient having undergone deep brain stimulation within the last 3 months or planning to have one during the year;
- Patient incapable of understanding the protocol, filling in a notebook or any other assessment criterion or of following the clinical trial procedures;
- Patient with cognitive decline that could interfere with the assessment;
- Patient under temporary guardianship/full guardianship/judicial protection.
  1. Number of subjects required

If we want a power of 80% for a difference of 4 points in quality of life (PDQ-39), given a standard deviation of 17 points and a level of 5% in a one-tailed test, we must recruit 111 Parkinson’s patients given a correlation coefficient of 50% between the answers for the two periods. Given the potential withdrawals from the trials, we will recruit 120 patients.

The number of subjects was calculated using data from a study using the PDQ-39 quality of life scale on Parkinson’s patients (Brefel-Courbon et al., 2003).

- 1. Recruitment methods

Patients with Parkinson’s disease will be selected at the Neurology consultation services at the 2 hospitals in Toulouse (Purpan and Rangueil) by neurologists specialized in caring for Parkinson’s patients (Dr. Brefel-Courbon, Dr. Fabre, Dr. Ory-Magne, Prof. Rascol) or at the movement disorder unit of the Neurology service at Purpan Hospital. This study seeks to be pragmatic, so we will propose this study consecutively and not selectively to all Parkinson’s patients meeting the inclusion and exclusion criteria.

We expect an average recruitment of 5 to 10 patients a month. The duration of inclusion should be 24 months.

- 1. Randomization

Randomization will be performed after the inclusion visit. It will assign the patients to one of the two groups: group I: patients following therapeutic education sessions and group II: patient receiving simple conventional medical monitoring. It will be done after verification of the inclusion criteria. It should lead to the inclusion of the number of patients needed in the study groups. This randomization will be carried out by the Centre d’Investigation Clinique (Clinical Investigation Center) at Purpan Hospital.

- 1. Conduct of the study

Each patient in the study will participate for 12 months. The total duration of the inclusion period is planned to last 2 years.

- 1. Inclusion visit

This will be done at a normal neurology follow-up consultation and will include:

- obtaining free, informed consent from the patient;

- verification of the inclusion and exclusion criteria;

- the patient’s motor score (UPDRS in the On condition for Parkinson’s patients);

- general and neurological medical examinations;

- completing the self-assessment questionnaires;

- distribution of the notebook and explanations as to how to fill it in;

- gathering the patient’s characteristics, disease data (case history, related comorbidities) and medication consumption.

All travel between the home and the hospital for pre-inclusion, inclusion and follow-up visits will be covered by the CPAM as these visits will be part of the follow-up visits for their chronic neurological disease.

### Follow-up and end-of-study visits

Patients will be assessed during a normal neurology follow-up consultation at 6 and 12 months after their inclusion.

During these 2 visits, the patient:

- will receive a neurological examination with a motor rating;
- fill in the self-assessment questionnaires;
- return the completed notebook and receive the notebook for the next visit (for the visit at 6 months).
  1. Data analysis

Data will be saved using the SAS software. The data input form will correspond exactly to the paper form used for gathering data.

The data will be made anonymous before its collation.

The statistical analysis will be descriptive as well as inferential:

- Qualitative variables will be described by presenting the frequency of each modality of these variables. Quantitative variables will be analyzed to establish the median, mean, standard deviation and extreme values.

- Quality of life will be analyzed classically using a linear model including a fixed treatment effect.

Comparisons of the medical costs, motor and psychological symptomatology (secondary quantitative assessment criteria) between two treatments will also be carried out in accordance with the process described above.

1. Expected consequences of the results of the project

If the impact of a TEP program is positive in terms of quality of life and if it does not produce additional costs, this type of care will be generalized for Parkinson’s disease and will give rise to contributions at national and international conferences.

1. Provisional schedule

Start of inclusions September 2008

End of inclusions September 2010

End of patient follow-up September 2011

Analysis of the results December 2011

1. Regulatory and ethical aspects

In accordance with law No. 2004-806 of 9 August 2004 relative to public health policy and its implementing orders, the project comes within the framework of an assessment of intermediate care and the study is subject to the opinion of a CPP (Comité de Protection des Personnes Sud-ouest et Outre-Mer – Institutional Review Board of the Southwest and Overseas Possessions). The research will not be implemented until a favorable opinion is received from the CPP.

All the information gathered is confidential and cannot be disclosed. The participants will be assured that the anonymity of each subject participating the study is guaranteed. No information that can be used to identify people will be transmitted to third parties other than those representing the sponsor and the Ministry of Health, who are authorized under the regulations to hold such information (and are bound by professional secrecy requirements).

Information gathered during this study will undergo data processing. An application for an authorization for automated data processing in research in the healthcare field will be filed with the CNIL (Commission Nationale de l'Informatique et des Libertés – French National Data Protection Authority).

Subjects will be informed of the study objectives and their rights to refuse participation. As this study comes under the framework of the assessment of intermediate care, its conduct and the subject’s participation or non-participation will in no way affect his/her usual care. Furthermore, in accordance with article 3 of the decree of 9 March 2007, the subjects will be informed of the study by a patient information letter and can manifest their opposition to participate in the study. They will be asked to sign a participation agreement.

After the start of the trial, any substantial modification thereto must be submitted to the study leader, who must obtain a favorable opinion from the CPP prior to implementation.

1. Bibliography

**-** Achard S, Chignon JM, Poirier-Littre MF, Galinowski A, Pringuey D, Van Os J, Lemonnier F. [Social adjustment and depression: value of the SAS-SR (Social Adjustment Scale Self-Report). Encephale. 1995;21:107-16.

- Brefel-Courbon C, Desboeuf K, Thalamas C, Galitzky M, Senard JM, Rascol O, Montastruc JL. Clinical and economic analysis of spa therapy in Parkinson's disease. Mov Disord. 2003;18:578-84.

**-** De Boer AGEM, Wijker W, Speelman JD, de Haes JCJM**.** Quality of life in patients with Parkinson’s disease: the development of a questionnaire*. J Neurol Neurosurg Psychiatry* 1996; 61: 70-74.

**-** Carrère MO. La qualité de vie liée à la santé et l’évaluation économique. Journal d’Economie Médicale 1997; 15: 5-8.

**-** Fahn S, Elton RL, and members of the UPDRS Development Committee: Unified Parkinson's disease rating scale. In: Recent developments in Parkinson's disease. (Eds Fahn S, Marsden CD, Calne DB, Goldstein M). Florham Park MacHillan Healthcare Information, 1987: 153-163.

**-** Fitzsimmons B, Bunting LB**.** Parkinson’s disease: quality of life issues. Neuroscience nursing 1993; 28:807-818.

**-** Gibb WK, Lees AJ**.** The relevance of the Lewy body to the pathogenesis of the idiopathic Parkinson's disease. *J Neurol NeuroSurg Psychiatry* 1988; 51: 745-752.

- [Grosset KA, Grosset DG.](http://www.ncbi.nlm.nih.gov/pubmed/17634109?ordinalpos=2&itool=EntrezSystem2.PEntrez.Pubmed.Pubmed_ResultsPanel.Pubmed_RVDocSum) Effect of educational intervention on medication timing in Parkinson's disease: a randomized controlled trial. BMC Neurol. 2007 16;7:20.

-Karlsen KH, Larsen JP, Tandberg E, Maeland JG. Influence of clinical and demographic variables on quality of life in patients with Parkinson’s disease. J Neurol Neurosurg Psychiatry 1999; 66: 431-435.

- Lépine JP, Godchau M, Brun P. Anxiety and depression in patients. Lancet, 1985, ii, 1425-1426.

- Macht M, Gerlich C, Ellgring H, Schradi M, Rusiñol AB, Crespo M, Prats A, Viemerö V, Lankinen A, Bitti PE, Candini L, Spliethoff-Kamminga N, de Vreugd J, Simons G, Pasqualini MS, Thompson SB, Taba P, Krikmann U, Kanarik E. Patient education in Parkinson's disease: Formative evaluation of a standardized programme in seven European countries. Patient Educ Couns. 2007; 65:245-52.

- [Mercer BS](http://www.ncbi.nlm.nih.gov/sites/entrez?Db=pubmed&Cmd=Search&Term="Mercer BS"%5BAuthor%5D&itool=EntrezSystem2.PEntrez.Pubmed.Pubmed_ResultsPanel.Pubmed_RVAbstractPlus). A randomized study of the efficacy of the PROPATH Program for patients with Parkinson disease. [Arch Neurol.](javascript:AL_get(this, 'jour', 'Arch Neurol.');) 1996 ;53 :881-4.

- [Montgomery EB Jr](http://www.ncbi.nlm.nih.gov/sites/entrez?Db=pubmed&Cmd=Search&Term="Montgomery EB Jr"%5BAuthor%5D&itool=EntrezSystem2.PEntrez.Pubmed.Pubmed_ResultsPanel.Pubmed_RVAbstractPlus), [Lieberman A](http://www.ncbi.nlm.nih.gov/sites/entrez?Db=pubmed&Cmd=Search&Term="Lieberman A"%5BAuthor%5D&itool=EntrezSystem2.PEntrez.Pubmed.Pubmed_ResultsPanel.Pubmed_RVAbstractPlus), [Singh G](http://www.ncbi.nlm.nih.gov/sites/entrez?Db=pubmed&Cmd=Search&Term="Singh G"%5BAuthor%5D&itool=EntrezSystem2.PEntrez.Pubmed.Pubmed_ResultsPanel.Pubmed_RVAbstractPlus), [Fries JF](http://www.ncbi.nlm.nih.gov/sites/entrez?Db=pubmed&Cmd=Search&Term="Fries JF"%5BAuthor%5D&itool=EntrezSystem2.PEntrez.Pubmed.Pubmed_ResultsPanel.Pubmed_RVAbstractPlus). Patient education and health promotion can be effective in Parkinson's disease: a randomized controlled trial. PROPATH Advisory Board. [Am J Med.](javascript:AL_get(this, 'jour', 'Am J Med.');) 1994; 97:429-35

**-** Petit H, Alain H, Vermersch P. La maladie de Parkinson, clinique et thérapeutique. Edition Masson, Paris, 1994, 7- 10.

- Rajendran PR, Thompson RE, Reich SG. The use of alternative therapies by patients with Parkinson’s disease. Neurology 2001; 57: 790-794.

- Riazi A, Hobart JC, Lamping DL, Fitzpatrick R, Freeman JA, Jenkinson C, Peto V, Thompson AJ. Using the SF-36 measure to compare the health impact of multiple sclerosis and Parkinson's disease with normal population health profiles. J Neurol Neurosurg Psychiatry. 2003; 74:710-4.

- Shimbo T, Goto M, Morimoto T, Hira K, Takemura M, Matsui K, Yoshida A, Fukui T. Association between patient education and health-related quality of life in patients with Parkinson's disease. Qual Life Res. 2004; 13:81-9.

- Schrag A, Jahanshahi M, Quinn N. How does Parkinson's disease affect quality of life? A comparison with quality of life in the general population. Mov Disord. 2000; 15:1112-8.

- [Simons G, Thompson SB, Smith Pasqualini MC; Members of the EduPark consortium.](http://www.ncbi.nlm.nih.gov/pubmed/16781881?ordinalpos=3&itool=EntrezSystem2.PEntrez.Pubmed.Pubmed_ResultsPanel.Pubmed_RVDocSum) An innovative education programme for people with Parkinson's disease and their carers. Parkinsonism Relat Disord. 2006; 12:478-85.

**-** Schipper H, Clinch JJ, Olweny CLM. Quality of Life Studies: definitions and conceptual issues. *Quality of Life and Pharmacoeconomics in Clinical Trials*. Second Edition, edited by B. Spilker. Lippincott-Raven Publishers, Philadelphia, 1996.

- Tison F. Maladie de Parkinson et syndromes parkinsoniens. In: La maladie de Parkinson. Acanthe, Masson, SmithKline Beecham, Paris, 1998: 41-61.

- Ware J, Sherbourne C. The MOS 36-item Short Health Survey 1: conceptual frame work and item selection. Med Care 1992; 30: 473-483.

1. APPENDIXES

Appendix 1: Patient information sheet and participation agreement

Appendix 2: Participation agreement

Appendix 3: Addendum to the patient information sheet

- 1. Appendix 1:

**STUDY**

**“ASSESSMENT OF A THERAPEUTIC EDUCATION PROGRAM FOR PARKINSON’S DISEASE.”**

**ETPARK Study.**

Dear Sir or Madam,

**The objective of this document to provide you with all the information you need to understand the purpose of this study, as well as the constraints and interest it may have for you. Please feel free to ask your physician to explain any words or expressions that you do not understand.**

You have just been hospitalized or have had a consultation at the Neurology Service of the CHU Toulouse University Medical Center for Parkinson’s disease. Your physician has proposed to have you take part in a study on the quality of care whose objective is to better understand this disease and to study how it is treated in order to ensure better care.

This study is being carried out in compliance with Law 2004-806 relative to public health policy (Articles L.1121-1 to 1126-7 of the Public Health Code). It has received a favorable opinion from the CPP (Comité Protection des Personnes Sud-Ouest et Ouest Mer II - Institutional Review Board of the Southwest and Overseas Possessions II) dated 6 November 2008*.*

This survey has no influence on medical prescriptions and care for your disease during your hospitalization nor on follow-up care over the longer term. Your attending physician is free to modify the treatment you receive if needed. Your participation in this study will last 1 year.

Antiparkinsonian treatments are only symptomatic and are sometimes insufficient. A therapeutic education program alongside the usual medicinal antiparkinsonian treatment could have a beneficial action on various components of the disease and thus improve the quality of life of Parkinson’s patients.

The objective of this study is to show an improvement in the quality of life of Parkinson’s patients following a therapeutic education program compared with Parkinson’s patients who do not follow such a program.

The Parkinson’s patients will be randomly assigned to two groups: the first group will follow a therapeutic education program for 12 months in combination with their usual antiparkinsonian treatment and the second group will receive the usual antiparkinsonian treatment without the therapeutic education program.

The therapeutic education program is designed to help patients to acquire or maintain the skills they need to manage their lives with a chronic disease as well as possible. The aim of this is to help them (as well as their families) to understand their disease and their treatment with a view to helping them to maintain and improve their quality of life. This program will include an initial consultation, then individual follow-up consultations (1 to 3 per three-month period), thematic group sessions (2 or 3 per six-month period), and then an assessment consultation at the end of the 12-month period.

In each group, we will assess quality of life using self-assessment questionnaires. These self-assessment questionnaires will be filled in at the start of the study and then at 6 and 12 months during a normal neurology follow-up consultation. It will take approximately 30 minutes to fill in the self-assessment questionnaires.

We will also gather figures for medical costs using a healthcare consumption notebook indicating your drugs, hospitalizations and consultations, which we ask you to fill in regularly at home.

If you agree to take part in this survey, no blood samples will be drawn other than those taken in the usual course of your treatment.

You are free to accept or refuse to participate in this research. Refusal to participate will have no consequences for your usual care.

You are free to withdraw from this study at any time without engaging any responsibility or suffering any prejudice for doing so.

All the results obtained in this research will remain confidential, in accordance with the rules laid down in the law of 1 July 1994 relative to the automatic processing of healthcare data. You have taken note that your right to access and rectification as laid down in France’s “Informatique et Libertés” law (*law No. 2004-801 of 6 August 2004 amending law No. 78-17 of 6 January 1978 relative to information technology, data files and individual liberties*) can be exercised at any time by contacting Doctor Brefel-Courbon, Neurology Service A, CHU Purpan, Toulouse, France (tel.: +33 (0)5 61 77 75 16).

Doctor Brefel-Courbon

Date of deliverance of this patient information sheet: …. / ………../…………….

- 1. Appendix 2: PARTICIPATION AGREEMENT

From Mr., Mrs. ………………………………..…………… (patient’s last name and first name)

Address: …..……………………………………………………………….…

Street: …………………………………………………………………………

Postal code: ………………………………….. City: …………………………………………

Telephone: Landline: ………………………………………………………………

Doctor …………………………….. proposed having me take part in a study titled:

**ASSESSMENT OF A THERAPEUTIC EDUCATION PROGRAM FOR PARKINSON’S DISEASE.**

**ETPARK Study.**

I have read the information above and fully understand it. I was given all the time necessary to ask all the questions I had concerning the study and answers were given for all my questions. The content and meaning of this information were carefully explained to me. I was told that I am free to accept or refuse this study. Likewise, I am free to withdraw from this study at any time without engaging any responsibility or suffering any prejudice for doing so. This will have no effect on my relations with my physician concerning my treatment and follow-up care.

**I freely agree to take part in this study under the conditions laid down in this document.**

My consent does not absolve the physicians of their responsibilities. I retain all my rights ensured under the law. I agree to allow the data recorded during this research to undergo data processing. I have taken note that my right to access and rectification as laid down in France’s “Informatique et Libertés” law (article 40) can be exercised at any time. These data concerning me personally will remain STRICTLY CONFIDENTIAL. I may at any time request additional information from Doctor Brefel-Courbon, Neurology Service A (tel. +33 (0)5 61 77 75 16). I have taken note that I will receive a copy of this signed consent form.

Signed at ……………………………….,

Date ……. / ……. / ……. Date ……. / ……. / …….

Patient’s signature Investigator’s signature

- 1. Appendix 3: Addendum to the Study’s Patient Information Sheet

**“Assessment of a Therapeutic Education Program for Parkinson’s Disease” – ETPARK Study**

Dear Sir or Madam,

You have agreed to take part in the clinical study, “Assessment of a Therapeutic Education Program for Parkinson’s Disease” for which Dr. Christine Brefel-Courbon is the study physician. You have already received an information form concerning this study and you have signed an informed consent form for participation in this study.

This addendum provides supplementary information on this study. One of the purposes of the study is to assess the medical cost figures that we are currently gathering using the healthcare consumption notebook that you fill in regularly at home. Furthermore, we will need to obtain your social security number, which will enable us to understand your expenses related to private practice care (medical consultations, physiotherapists, speech therapy, etc.) recorded at the CPAM (Caisse Primaire d'Assurance Maladie – France’s Primary Health Insurance Fund) and your expenses for hospitalizations. This will be done for the protocol period only. This information will be gathered anonymously and your social security number will not be used for any other purpose.

You are not required to take part in this study supplement to pursue your participation in the study.

I, the undersigned, Last name: …………………..…First name: …………………….....,

after having read this addendum to the patient information sheet and having discussed and obtained answers to all my questions:

- authorize access to my social security number __

- do not authorize access to my social security number __

Signed at …………………………..

Date ……. / ……. / ……. Date ……. / ……. / …….

Patient’s signature Investigator’ signature

*Signed in triplicate: one copy is given to the volunteer, the second copy is kept by the investigator,*

*the third is for the sponsor.*
