# Supplementary material for: Clinical Impact and Cost-Effectiveness of an Education Program for PD Patients: A Randomized Controlled Trial
Source: PLoS One. 2016 Sep 29;11(9):e0162646. doi: 10.1371/journal.pone.0162646 (PMC5042480; doi:10.1371/journal.pone.0162646)
Supplement: S1 Table — (DOCX) [file pone.0162646.s006.docx]

**S1- Table:** Baseline status of SF36 scale of the TTBI and no TTBI groups.

|  | TTBI  (n=60) | no TTBI  (n=60) |
| --- | --- | --- |
| SF36  Physical functioning  Role, physical  Bodily discomfort  General health  Vitality  Social functioning  Role, emotional  Mental health | 75·0±25·9  43·1±38·3  68·4±30·8  50·5±22·3  51·3±18·7  78·8±21·5  62·1±41·3  63·7±21·8 | 73·1±19·3  28·0±32·1*  64·4±31·7  49·3±17·3  51·2±19·8  76·9±19·9  57·5±41·6  63·8±18·7 |

Values are means±SD

*: p<0.05
