# Supplementary material for: Clinical Impact and Cost-Effectiveness of an Education Program for PD Patients: A Randomized Controlled Trial
Source: PLoS One. 2016 Sep 29;11(9):e0162646. doi: 10.1371/journal.pone.0162646 (PMC5042480; doi:10.1371/journal.pone.0162646)
Supplement: S2 Table — (DOCX) [file pone.0162646.s007.docx]

**S2-Table**: Comparison of the changes (between the 12 months and baseline) of quality of life (SF 36) in the 2 groups

|  | TTBI  (*n*=60) | no TTBI  (*n*=60) | *P*  (non adjusted) | *P*  (adjusted) | Difference of not TTBI vs TTBI (95% CI) |
| --- | --- | --- | --- | --- | --- |
| SF36  Physical functioning  Role, physical  Bodily discomfort  General health  Vitality  Social functioning  Role, emotional  Mental health | -0.59±21.58  -4.46±35.63  -1.63±26.88  -2.33±17.24  3.00±19.36  6.78±20.28  4.1±45.46  4.55±20.07 | -2.33±11.97  -0.91± 26.33  -4.31± 28.72  0.51±15.93  -1.25±17.16  -0.43±17.67  -1.81±49.46  -1.53±15.06 | 0·59  0·55  0·60  0·35  0·20  0·04  0·51  0·06 | 0·43  0·41  0·43  0·34  0·33  0·01  0·63  0·19 | -1.73 (-8.14 ; 4.67)  3.56 (-8.25 ; 15.36)  -2.67 (-12.77 ; 7.43)  2.83 (-3.19 ; 8.86)  -4.25 (-10.86 ; 2.36)  -7.20 (-14.13 ; -0.27)  -5.92 (-23.70 ; 11.76)  -6.08 (-12.50 ; 0.33) |

Values are means±SD ; p is adjusted on disease duration
