# Supplementary material for: Clinical Impact and Cost-Effectiveness of an Education Program for PD Patients: A Randomized Controlled Trial
Source: PLoS One. 2016 Sep 29;11(9):e0162646. doi: 10.1371/journal.pone.0162646 (PMC5042480; doi:10.1371/journal.pone.0162646)
Supplement: S3 Table — (DOCX) [file pone.0162646.s008.docx]

**S3-Table**: Comparison of the changes (between the 6 months and baseline) of quality of life UPDRS and psychological scores.

|  | TTBI  (*n*=60) | no TTBI  (*n*=60) | *P*  (non adjusted) | *P*  (adjusted) | Difference of no TTBI vs TTBI (95% CI) |
| --- | --- | --- | --- | --- | --- |
| PDQ-39  Mobility  ADL  Emotional well being  Stigma  Social Support  Cognition  Communication  Bodily discomfort | -1.21.±12.55  -2.07±12.96  -6.10±16.14  -4.75±17.42  1.41±12.20  -1.58±11.46  -0.85±12.83  -3.05±13.72 | 0.00±10.37  1.93±12.96  -3.09±14.26  -5.51±16.30  -1.32±12.37  -0.01±13.68  -0.98±14.69  -1.43±15.23 | 0.58  0.12  0.29  0.81  0.23  0.50  0.95  0.54 | 0.51  0.14  0.37  0.77  0.25  0.64  0.79  0.68 | 1.21(-3.07 ; 5.49)  4.00 (-1.06 ; 9.06)  3.00 (-2.60 ; 8.61)  -0.76 (-6.94 ; 5.42)  -2.73 (-7.25 ; 1.79)  1.58 (-3.05 ; 6.20)  -0.13 (-5.16 ; 4.90)  1.62 (-3.66 ; 6.90) |
| UPDRS  UPDRS I  UPDRS II  UPDRS III  UPDRS IV  Total score | -0.72±1·28  -1.32±2.51  -1.28±4.20  -0.12±1.80  -3.43±6.45 | 0.05±1.40  0.65±2.29  1.35±4.19  0.17±1.72  2.22±5.67 | <0.01  <0·0001  <0·001  0·38  <0.0001 | 0.01  <0.0001  <0·01  0·26  <0.0001 | 0.77 (0.28 ; 1.25)  1.97 (1.10 ; 2.83)  2.63 (1.11; 4.15)  0.28 (-0.35 ; 0.92)  5.65 (3.45 ; 7.75) |
| Anxiety and Depression  Anxiety  Depression  Total score | -1.12±3.30  -0.62±2.58  -1.73±4.68 | -0.57±2.60  -0.50±3.13  -1.07±4.83 | 0·31  0·82  0·44 | 0·51  0·88  0·61 | 0.55 (-0.53 ; 1.63)  0.12 (-0.92 ; 1.16)  0.67 (-1.05 ; 2.39) |
| SF36  Physical functioning  Role, physical  Bodily discomfort  General health  Vitality  Social functioning  Role, emotional  Mental health | 2.00±15.66  -0.26±35.96  0.10±28.33  -0.82±12.63  2.25±17.79  4.22±20.68  0.57±45.28  3.95±18.06 | -3.25±13.11  5.19± 29.97  -1.52± 33.05  -0.85±16.14  1.25±16.25  -1.06±21.31  12.50±48.23  4.27±14.21 | 0·05  0·38  0·77  0·99  0·74  0·18  0·17  0·91 | 0·04  0·28  0·40  0·96  0·82  0·11  0·35  0·60 | -5.25 (-10.55 ; 0.05)  5.45 (-7.07 ; 17.97)  -1.62 (-12.80 ; 9.54)  -0.03 (-5.33 ; 5.28)  -1.00 (-7.16 ; 5.16)  -5.28 (-12.97 ; 2.41)  -11.93 (-5.35 ; 29.21)  0.32 (-5.58 ; 6.22) |

Values are means±SD ; p is adjusted on disease duration
